# A rank-based normalization method with the fully adjusted full-stage procedure in genetic association studies

**DOI:** 10.1371/journal.pone.0233847

**Published:** 2020-06-19

**Authors:** Li-Chu Chien

**Affiliations:** Center for Fundamental Science, Kaohsiung Medical University, Kaohsiung, Taiwan; Brigham and Women's Hospital and Harvard Medical School, UNITED STATES

## Abstract

In the area of genetic epidemiology, studies of the genotype-phenotype associations have made significant contributions to human complicated trait genetics. These studies depend on specialized statistical methods for uncover the association between traits and genetic variants, both common and rare variants. Often, in analyzing such studies, potentially confounding factors, such as social and environmental conditions, are required to be involved. Multiple linear regression is the most widely used type of regression analysis when the outcome of interest is quantitative traits. Many statistical tests for identifying genotype-phenotype associations using linear regression rely on the assumption that the traits (or the residuals) of the regression follow a normal distribution. In genomic research, the rank-based inverse normal transformation (INT) is one of the most popular approaches to reach normally distributed traits (or normally distributed residuals). Many researchers believe that applying the INT to the non-normality of the traits (or the non-normality of the residuals) is required for valid inference, because the phenotypic (or residual) outliers and non-normality have the significant influence on both the type I error rate control and statistical power, especially under the situation in rare-variant association testing procedures. Here we propose a test for exploring the association of the rare variant with the quantitative trait by using a fully adjusted full-stage INT. Using simulations we show that the fully adjusted full-stage INT is more appropriate than the existing INT methods, such as the fully adjusted two-stage INT and the INT-based omnibus test, in testing genotype-phenotype associations with rare variants, especially when genotypes are uncorrelated with covariates. The fully adjusted full-stage INT retains the advantages of the fully adjusted two-stage INT and ameliorates the problems of the fully adjusted two-stage INT for analysis of rare variants under non-normality of the trait. We also present theoretical results on these desirable properties. In addition, the two available methods with non-normal traits, the quantile/median regression method and the Yeo-Johnson power transformation, are also included in simulations for comparison with these desirable properties.

## Introduction

In recent years, there has been growing interest in using next-generation sequencing technologies to discovery causal rare variants associated with complex human disease and traits. Association studies, where the correlational relationship between genetic variants and traits are evaluated, are helpful for mapping genes influencing complex diseases. In the area of genetic epidemiology, the genotype-phenotype associations of genetic markers with quantitative traits of interest are typically tested through liner regression under the assumption of the normality and finite variance for the trait distribution [1, 2]. However, in practical applications, the true model is unknown and thus the assumption of normally distribution with finite variance in samples of sufficient size may be violated [2, 3]. The operating parameters of linear regression are sensitive to the underlying trait distributions and outliers [2]. Ignoring outliers or non-normality can seriously affect the type I error rates and statistical power, which especially leads to worse impact on rarer variants [4, 5].

In genetic association studies, the rank-based inverse normal transformation (INT) to the phenotype is widely used as a direct manner to fulfill the assumption of normality of the outcome [3, 6–8]. Regardless of the underlying trait distribution, the distribution of the trait after the INT is expected to be normal [2]. Many genetic researchers believe that such INT transformations are necessary for valid inference especially in studying rare-variant associations [8]. For example, Tang and Lin [9] showed that applying the INT to the trait values can ameliorate the type I error rates and enhances statistical power in detecting associations relative to rare-variant analyses. However, in many situations, the use of the phenotype transformation has been demonstrated to be insufficient for normalizing data. For example, Sofer et al. [5] pointed out that affecting the valid statistical inference of regression-based genotype-phenotype association tests is not the distribution of the trait but the distribution of the trait after regressing out covariates. Pain et al. [10] indicated that the INT always make a perfect normal distribution when no tied observations exist in the dataset. Previous researches have exhibited that albeit the INTs give rise to potential loss of information, this approach keeps good control of type I error rate and statistical power [10–12]. Beasley et al. [3] reported that the applying the INT to traits may still lead to non-normal residuals and then result in the improper type I error control under certain circumstances where the residuals follow a heavily skewed distribution.

In recent years, genome-wide association studies (GWAS) have been analyzed by the two-stage INT approach. In Stage 1, the INT approach is applied to the residuals that are obtained by regressing the traits on covariates and afterward these INT-transformed phenotypic residuals are used to be regressed on genotype without further adjustment for covariates in Stage 2 [13–17]. It is called the partly adjusted two-stage INT. The properties of this frequently used method has been investigated by Che et al. [18] and Demissie and Cupples [19] that found out that the partly adjusted two-stage INT has undesirable statistical properties, such as the bias of the estimates, power and type I error rates, under the situation with the correlational relationship between covariates and genotypes. Pain et al. [10] discussed that these unsuitable statistical properties are a consequence of the INT of the phenotypical residuals re-introducing a correlational relationship in the opposite direction between the covariates and the rank-normalized phenotypic residuals. Sofer et al. [5] showed that such a partly adjusted two-stage INT results in these undesirable statistical properties because of a mis-specified mean-variance relationship for the genetic effect. To address these issues, Sofer et al. [5] further introduced a modification version of the partly adjusted two-stage INT, which is called the fully adjusted two-stage INT. In Stage 1, processing the same procedure as Stage 1 of the partly adjusted two-stage INT, they used the INT to rank-normalize the phenotypic variable after regressing out covariates and then obtain the INT-transformed phenotypic residuals. However, in Stage 2, they run a regression of these rank-normalized phenotypic residuals on the genotypes with adjusting for the same covariates used in Stage 1. Sofer et al. [5] showed that the fully adjusted two-stage INT approach improves these undesirable statistical properties of the partly adjusted two-stage INT approach for analysis of rare variants.

On the other hand, McCaw et al. [2] proposed the INT-based omnibus test (O-INT) that systematically combine the direct (D-INT) and indirect (I-INT) INT-based association tests. In the direct method (D-INT), the phenotypes are first transformed to normality using the INT procedure and then the INT-transformed phenotypes are simultaneously regressed on genetic factors and covariates. In the indirect method (I-INT), the INT procedure is applied to the residuals that are obtained by regressing the phenotypes on covariates and then these INT-transformed residuals are regressed on genetic factors with or without the adjustment for covariate effects (e.g., population structure). McCaw et al. [2] showed that the O-INT test is more robust and powerful than the existing INT tests, for the analysis of GWAS of quantitative traits with non-normally distributed residuals.

In addition, some available methods with non-normal traits (or non-normal residuals) had been successfully applied to some specific objectives in genetic analysis. For example, the quantile regression method [20] had been used to analyze GWAS data in human genetics [21] and in flowering time-related traits in common bean [22]. On the other hand, the Box-Cox power transformation [23] had been applied to omics data [24]. Moreover, the Yeo-Johnson power transformation [25] had been utilized to analyze to the gene expression data [26].

However, as discussed by Sofer et al. [5], some researchers (e.g., Auer et al. [4]) reported that the INT-based technique still has its advantage of the rare variant analysis in practice. Moreover, detecting rare variants in complex diseases via whole-genome sequencing is a hot topic in genetic association analysis. Hence it is necessary to investigate how transformations and covariate-variant relationships interact to impact on genetic effects and to provide a comprehensive framework for studying genetic association analysis for rare variants with quantitative traits using the INT-based procedures.

In this investigation, we propose a test by using a fully adjusted full-stage INT approach for detecting the association of rare (and common) variants with a quantitative trait under the situations with departure of the trait distribution from normality. More precisely, we propose a fully adjusted full-stage INT method that keeps the merits of the fully adjusted two-stage INT approach that provides the preservation of the fundamental core of the INT and alleviates the potentially incorrect inference arose from the partly adjusted two-stage INT approach in analysis of both common and rare variants [5]. Maintaining these desirable merits of the fully adjusted two-stage INT approach, the proposed full-stage INT approach further assuages the potential for incorrect inference arose from the fully adjusted two-stage INT approach in analysis of rare variants, especially when the SNP (genetic) effects are unrelated to covariates.

The remainder of this paper is organized in the following way. In the materials and methods section, we present the existing INT-based methods, the partly and fully adjusted two-stage INT methods, and further propose the fully adjusted full-stage INT approach that can help control type I error inflation arose from the existing INT-based methods. In the simulation studies, we exhibit evidence that the proposed full-stage INT method is more robust than the exiting INT approaches in controlling the type I error rates under the situation with the genotypes that are uncorrelated with covariates. Simultaneously the proposed full-stage INT method has good control of power in rare variant association analysis, as the fully adjusted two-stage INT method. We present theoretical results on these desirable properties in Appendix. In addition, the two available methods with non-normal traits, the median regression method and the Yeo-Johnson power transformation, are also included in simulations for comparison with these desirable properties.

## Materials and methods

To describe the fully adjusted full-stage INT approach, in this section we first present the existing methods of the fully adjusted two-stage INT procedure introduced by Sofer et al. [5] and the partly adjusted two-stage INT procedure that is now widely used in genome-wide association studies. Then we explain how to improve the idea of the fully adjusted two-stage INT approach and then propose a fully adjusted full-stage INT approach. Furthermore, we illustrate how to identify the association between the rare variants and traits by using the fully adjusted full-stage INT procedure and explain its advantages.

### Setting

We consider a sample with *n* independent individuals. Suppose that for each of *n* independent individuals, we have a continuous (quantitative) trait *y*_*i*_, *g*_*i*_ = 0,1 or 2 is the genotype score for a single nucleotide polymorphism (SNP) of interest, and ***x***_*i*_ = (*x*_*i*,0_,*x*_*i*,1_,⋯,*x*_*i*,*p*−1_)^*T*^ is a *p*×1 vector of covariates (confounding factors) with the intercept term *x*_*i*,0_ = 1, which are considered to be adjusted for. For convenience of notation, let ***y*** = (*y*_1_,*y*_2_,⋯,*y*_*n*_)^*T*^ denote the *n*×1 vector of the observed traits over *n* observations. Correspondingly, ***g*** = (*g*_1_,*g*_2_,⋯,*g*_*n*_)^*T*^ stands for the *n*×1 vector of the observed genotypes and ***X*** = (***x***_1_,***x***_2_,⋯,***x***_*n*_)^*T*^ represents the *n*×*p* design matrix corresponding to the covariate effects.

### Multiple linear regression

In the multiple regression model, the relationship between ***y***, ***X*** and ***g*** is given by

y=Xα+gβ+ε (1)

where ***α*** = (*α*_0_,*α*_1_,⋯,*α*_*p*−1_)^*T*^ is a *p*×1 vector of regression coefficients of the covariates, *β* is the regression coefficient of the SNP genotype, ***ε*** = (*ε*_1_,*ε*_2_,⋯,*ε*_*n*_)^*T*^ is an *n*×1 vector of random errors with each component independently from *N*(0,*σ*^2^), the normal distribution with a mean of zero and a variance of *σ*^2^. Here the main focus is to examine the null hypothesis that there is no association between the SNP genotype and the trait component. According to Eq (1), the null hypothesis of no association between ***g*** and ***y*** is *H*_0_:*β* = 0 [18, 19]. The Wald statistic and the likelihood ratio statistic are frequently employed for testing *H*_0_:*β* = 0 with estimates based on the least squares method [2, 18, 19]. Another method frequently used for testing *H*_0_:*β* = 0 is the score statistic that is based on the residual obtained by regressing the trait on the covariates [2, 5]. As mentioned in Sofer et al. [5], a score statistic widely applied in genetic association analysis, for example, is the sequence kernel association test (SKAT, [27]).

### The fully adjusted two-stage INT approach

In the first stage of the fully adjusted two-stage INT approach proposed by Sofer et al. [5], the (raw) residuals $\hat{\varepsilon }=y-X\hat{\alpha }={\left(\varepsilon_{1},\varepsilon_{2},\cdots ,\varepsilon_{n}\right)}^{T}$ under the null hypothesis of *H*_0_:*β* = 0 are obtained by regressing the traits ***y*** on the covariate matrix ***X*** with the estimate of the covariate effects $\hat{\alpha }$ calculated through the least squares method. Then the INT-transformed residuals, $\mathrm{RN}({\hat{\varepsilon }}_{i}),i=1,2,\cdots n,$ are obtained by applying the INT procedure to the residuals, ${\hat{\varepsilon }}_{i},i=1,2,\cdots ,n,$ namely,

$$\mathrm{RN}({\hat{\varepsilon }}_{i})=\Phi^{-1}\left\{\frac{\mathrm{rank}({\hat{\varepsilon }}_{i})-c}{n}\right\},c\in [0,1/2],\;\mathrm{for}\;i=1,2,\cdots ,n$$ (2)

where $\mathrm{rank}({\hat{\varepsilon }}_{i})$ is the rank of the *i*th observation among the *n* residuals and Φ^−1^ stands for the standard normal quantile function. The INT-transformed residuals, $\mathrm{RN}({\hat{\varepsilon }}_{i}),i=1,2,\cdots ,n,$ in Eq (2) independently follow the standard normal distribution and retain the same rank as the residuals, ${\hat{\varepsilon }}_{i},i=1,2,\cdots ,n$ ([9],[5]). In the second stage of a fully adjusted two-stage INT approach, the INT-transformed residuals are regressed on the SNP genotype and the covariate matrix that is adjusted in the first stage in order to examine the association between the INT-transformed residuals and the SNP genotype. When the covariate matrix only includes the intercept that is adjusted in the second stage, such a process is called the partly adjusted two-stage INT approach. Sofer et al. [5] theoretically showed that without a rank-normalization for transforming the (raw) residuals, ${\hat{\varepsilon }}_{i},i=1,2,\cdots ,n,$ considered, the partly adjusted two-stage approach in which the (raw) residuals, ${\hat{\varepsilon }}_{i},i=1,2,\cdots ,n,$ in the second stage are regressed on the genotypes without further adjustment for the covariates causes type I error deflation and a disastrous loss in statistical power, whereas the fully adjusted two-stage INT approach in which the (raw) residuals, ${\hat{\varepsilon }}_{i},i=1,2,\cdots ,n,$ in the second stage are regressed on the genotypes and the same covariates as used in the first stage can well control type I errors and improve statistical power.

However, we note that the fully adjusted two-stage INT approach can result in tests with desirable statistical properties. It requires a strong assumption in the second stage. More precisely, the INT-transformed residuals, $\mathrm{RN}({\hat{\varepsilon }}_{i}),i=1,2,\cdots n,$ in the second stage are required to follow a normal distribution with a mean of zero and finite variance. Nevertheless, in practice, the INT-transformed residuals, $\mathrm{RN}({\hat{\varepsilon }}_{i}),i=1,2,\cdots n,$ in the second stage may not have a normal distribution in a two-stage procedure. Therefore, we attempt to propose a full-stage procedure for improving a two-stage procedure, when the assumption of a normal distribution that is applied to the INT-transformed residuals, $\mathrm{RN}({\hat{\varepsilon }}_{i}),i=1,2,\cdots n,$ in the second stage is violated.

### The fully adjusted full-stage INT approach

As have been mentioned by Pain et al. [10], previous investigations have shown that the INT approach has desirable performance on power and type I error rates, even if the INT approach maybe simplify and lose information from data in the transformation process [11, 12]. Therefore, we intend to again use the INT processes for normalizing the INT-transformed residuals, $\mathrm{RN}({\hat{\varepsilon }}_{i}),i=1,2,\cdots n,$ in the second stage in order to make the INT-transformed residuals follow a standard normal distribution, when the INT-transformed residuals, $\mathrm{RN}({\hat{\varepsilon }}_{i}),i=1,2,\cdots n,$ in the second stage doesn’t meet the assumption of a normal distribution with zero mean and finite variance. Extending such an idea, we further propose a fully adjusted full-stage INT approach for genetic association analysis. The fully adjusted full-stage INT approach not only maintains the merits of the fully adjusted two-stage INT approach but also ameliorates the defect of the fully adjusted two-stage INT approach. The algorithm of the fully adjusted full-stage INT approach is given below.

Stage 1. Calculate the (raw) residuals $\hat{\varepsilon }={\left({\hat{\varepsilon }}_{1},{\hat{\varepsilon }}_{2},\cdots ,{\hat{\varepsilon }}_{n}\right)}^{T}=y-X\hat{\alpha }$ under the null hypothesis of *H*_0_:*β* = 0 through the R package *SKAT* [28], which has the same idea as that introduced in the first stage of the partly and fully two-stage INT manners.

Stage 2. Obtain the INT-transformed residuals, $\mathrm{RN}({\hat{\varepsilon }}_{i}),i=1,2,\cdots n,$ by employing the INT procedure for transforming the residuals, ${\hat{\varepsilon }}_{i},i=1,2,\cdots ,n,$ namely,

$$\mathrm{RN}({\hat{\varepsilon }}_{i})=\Phi^{-1}\left\{\frac{\mathrm{rank}({\hat{\varepsilon }}_{i})-c}{n}\right\},\;\mathrm{for}\;i=1,2,\cdots ,n$$

where we choose the conventional offset of *c* = 1/2 [4, 29].

Stage 3. Regress the INT-transformed residuals, $\mathrm{RN}({\hat{\varepsilon }}_{i}),i=1,2,\cdots n,$ on the covariate matrix ***X*** by using the R package *glm* and obtain the *p*-values of the covariate effects. If one of *p*-values of the covariate effects is less than 0.05, then go to Stage 4. Otherwise go to Stage 5.

Stage 4.

Step 1. Regress the INT-transformed residuals, $\mathrm{RN}({\hat{\varepsilon }}_{i}),i=1,2,\cdots n,$ on the covariate matrix ***X*** by using the R package *glm* and obtain the estimates of the covariate effects $\tilde{\alpha }.$

Step 2. Calculate the residual ${\hat{\varepsilon }}^{*}={\left({\hat{\varepsilon }}_{1}^{*},{\hat{\varepsilon }}_{2}^{*},\cdots ,{\hat{\varepsilon }}_{3}^{*}\right)}^{T}$ by regressing the INT-transformed residuals, $\mathrm{RN}({\hat{\varepsilon }}_{i}),i=1,2,\cdots n,$ on the covariate matrix ***X*** and obtain the INT-transformed residuals $\mathrm{RN}({\hat{\varepsilon }}^{*}{}_{i})=\Phi^{-1}\left\{\left(\mathrm{rank}({\hat{\varepsilon }}_{i}^{*})-0.5\right)/n\right\},\;\mathrm{for}\;i=1,2,\cdots ,n.$ Then the INT-transformed residuals, $\mathrm{RN}({\hat{\varepsilon }}_{i}^{*}),i=1,2,\cdots n,$ are regressed on the covariate matrix ***X*** by using the R package *glm* and obtain the *p*-values and estimates of the covariate effects denoted by p* and α*, respectively.

Step 3. Re-define $\mathrm{RN}({\hat{\varepsilon }}_{i})$ by $\mathrm{RN}({\hat{\varepsilon }}_{i}^{*})$ substituted for $\mathrm{RN}({\hat{\varepsilon }}_{i}),\;\mathrm{for}\;i=1,2,\cdots ,n.$ If all elements of *p*-values of the covariate effects, p*, are not less than 0.05 or the difference between the covariate effects $\tilde{\alpha }$ and α* is less than 10^−6^, then go to Stage 5. Otherwise repeat the above Steps 1–2 in Stage 4 and then repeat Step 3 in Stage 4.

Stage 5. Regress the INT-transformed residuals, $\mathrm{RN}({\hat{\varepsilon }}_{i}),i=1,2,\cdots n,$ on the SNP genotype ***g*** and the covariate matrix ***X*** by using the R package *SKAT* [28] and then obtain the *p*-value of the SNP (genetic) effect.

Evidently, the fully adjusted two-stage INT approach proposed by Sofer et al. [5] is a special case of the fully adjusted full-stage INT approach. When the INT-transformed residuals, $\mathrm{RN}({\hat{\varepsilon }}_{i}),i=1,2,\cdots n,$ in Stage 2 follow a normal distribution with zero mean and finite variance, only Stages 1–2 and Stage 5 of the fully adjusted full-stage INT method are used for testing the SNP (genetic) effect, which in turn means that the fully adjusted full-stage INT approach is simply reduced to the fully adjusted two-stage INT approach. On the other hand, when the INT-transformed residuals, $\mathrm{RN}({\hat{\varepsilon }}_{i}),i=1,2,\cdots n,$ in the second stage may not follow a normal distribution with zero mean and finite variance, we intend to ameliorate the INT-transformed residuals, $\mathrm{RN}({\hat{\varepsilon }}_{i}),i=1,2,\cdots n,$ in the second stage and make them have zero mean and one standard deviation through repetitively processing Stage 4 in the fully adjusted full-stage INT procedure.

A full-stage INT procedure in which the INT-transformed residuals $\mathrm{RN}({\hat{\varepsilon }}_{i}),i=1,2,\cdots n,$ are repeatedly improved by inverse normal transformations (INTs) until their distributions follow the normal distributions with zero mean and one standard deviation. Such a full-stage INT procedure leads to a robust control of the type I error specially under the situation in which genotypes are uncorrelated with covariates. In S1 Appendix, a mathematical detail for the Wald test statistic in a fully adjusted full-stage INT procedure is provided for explaining how to use the fully adjusted full-stage INT procedure for transforming the INT-transformed residuals, $\mathrm{RN}({\hat{\varepsilon }}_{i}),i=1,2,\cdots n,$ in the second stage of the fully adjusted two-stage INT method in order to make the INT-transformed residuals have a normal distribution with zero mean and one standard deviation, when the INT-transformed residuals, $\mathrm{RN}({\hat{\varepsilon }}_{i}),i=1,2,\cdots n,$ in the second stage don’t follow the assumption of a normal distribution with zero mean and finite variance. A similar result for the SKAT test based on the fully adjusted full-stage INT procedure can be obtained. In S2 Appendix, a mathematical detail for the partial *F* test in a fully adjusted full-stage INT procedure is provided for explaining why the fully adjusted full-stage INT procedure has a robust performance on control of the type I error specially under the situation in which genotypes are uncorrelated with covariates, in comparison with the fully adjusted two-stage INT procedure.

#### Simulation studies

We carry out numerical simulation studies to assess the finite sample performance of the proposed method, the fully adjusted full-stage INT method. We imitate the similar set-up as those described in the paper of Auer et al. [4], Sofer et al. [5] and McCaw et al. [2], with modification to investigate the effect of the INT technique for mitigating the potentially mis-calibrated inference. Seven existing methods, the median regression (MR) method, the Yeo-Johnson power transformation (YJPT) method, the SKAT test, the D-INT test, the I-INT test, the O-INT test and the fully adjusted two-stage INT method are included in our simulations for comparison. Here the MR method is a special case of the quantile regression when estimating the 0.5 quantile. The YJPT method can be used without restrictions on traits and retains the advantages of the Box-Cox power transform [30]. The MR method is implemented by the R package *rq* [31]. The YJPT method is implemented by the R package *car* [32]. The SKAT test is proposed by Wu et al. [27] and is implemented by the R package *SKAT* [28]. The D-INT, I-INT and O-INT methods are executed by the R package *RNOmni* [33].

### Evaluation of type I error rate and power

We sample quantitative traits according to the linear model

$$y_{i}=x_{i1}\alpha_{1}+x_{i2}\alpha_{2}+g_{i}\beta +\varepsilon_{i}$$

where the error terms *ε*_*i*_ are considered to be generated from three different types of distribution settings. First, normal error terms are considered. The error terms *ε*_*i*_ are sampled from the normal distribution having zero mean with the standard deviation of 1 and 0.01, respectively, considered. Secondly, the outliers involved in the error terms are considered. The error terms with the probability of 0.99 are sampled form the normal distribution with zero mean and a standard deviation of 0.01 and with the probability of 0.01 are sampled from the normal distribution with zero mean and a standard deviation of 3. Thirdly, non-normal error terms are considered. The error terms *ε*_*i*_ are sampled from the chi-squared distribution with two degrees of freedom. Here continuous covariates *x*_*i*1_ are sampled from a standard normal distribution. Binary covariates *x*_*i*2_ are sampled with an equal probability of being 0 or 1. The covariate effects *α*_1_ and *α*_2_ are set by 0.5.

On the other hand, as in Sofer et al. [5], the SNP genotype for each individual is generated from a binomial distribution with parameters *N* = 2 (traits) and probability *p*_*i*_ given by *p*_*i*_ = exp(*γ*_0_+*x*_*i*1_*γ*_1_)/(1+exp(*γ*_0_+*x*_*i*1_*γ*_1_)). Here *γ*_0_ is considered by -7, -4.5 and -2, respectively, whereas *γ*_1_ is considered by 0, 1 and 2, respectively. The value of *γ*_1_ is zero, which means that there is no correlation between the SNP genotype *g*_*i*_ and covariate *x*_*i*1_, whereas the value of *γ*_1_ is one or two, which means that there is a correlation between the SNP genotype *g*_*i*_ and covariate *x*_*i*1_. When *γ*_0_ = −7 and *γ*_1_ = 0, the value of *p*_*i*_ is 0.0009, which means the SNP genotype has a lower minor allele frequency (MAF) of 0.0009. When *γ*_0_ = −2 and *γ*_1_ = 0, the value of *p*_*i*_ is 0.1192, which means the SNP genotype has a larger MAF of 0.1192.

For type I error simulations, each combination of the parameter settings for *γ*_0_ and *γ*_1_ is carried out by the 10^6^ simulations with the SNP (genetic) effect *β* = 0 under the null hypothesis of no association between the SNP genotype *g*_*i*_ and the traits *y*_*i*_. For power simulations, each combination of the parameter settings for *γ*_0_ and *γ*_1_ is executed based on the 2×10^5^ simulations with the SNP (genetic) effect *β* set by 0.0012. Based on the sample size *n* = 2000 and 10000 respectively considered, empirical type I error rates and power rates at the nominal level of 0.0001 are reported for all simulation results.

## Results

### Empirical type I error rates

Tables 1 and 2 exhibit the comparison results of empirical type I error rates when the error terms *ε*_*i*_ are generated from the normal distribution with zero mean and the standard deviation of 1 and 0.01, respectively. Table 1 shows that the seven methods, the YJPT method, the SKAT test, the D-INT test, the I-INT test, the O-INT test and the fully adjusted two- and full-stage INT methods well control type I errors when the error terms follow a standard normal distribution. On the other hand, the MR method has inflated type I error rates when the sample size *n* is insufficiently large or the SNP genotype has a smaller MAF. A similar result obtained at the nominal level of 0.001 is presented in S1 Table.

**Table 1:** Empirical type I errors for the eight competing methods for each study at nominal level of 0.0001 based on error terms from a normal distribution with zero mean and a standard deviation of 1.

Sample	How	Con-								
Size	rare	founding	Association method							
*n*	*γ*_0_	*γ*_1_	MR^1^	YJPT^2^	SKAT^3^	D-INT^4^	I-INT^4^	O-INT^4^	TS-INT^5^	FS-INT^6^
2000	-7	0	0.00006	0.00011	0.00010	0.00009	0.00008	0.00008	0.00008	0.00008
		1	**0.03120**^†^	0.00011	0.00010	0.00009	0.00009	0.00009	0.00009	0.00009
		2	**0.00907**	0.00011	0.00010	0.00008	0.00010	0.00010	0.00011	0.00011
	-4.5	0	**0.00261**	0.00011	0.00011	0.00010	0.00011	0.00010	0.00011	0.00011
		1	**0.00326**	0.00013	0.00012	0.00011	0.00012	0.00011	0.00012	0.00012
		2	**0.00108**	0.00012	0.00011	0.00009	0.00011	0.00010	0.00011	0.00011
	-2	0	**0.00027**	0.00010	0.00009	0.00009	0.00009	0.00009	0.00009	0.00009
		1	**0.00017**	0.00011	0.00010	0.00010	0.00011	0.00010	0.00011	0.00011
		2	**0.00016**	0.00011	0.00010	0.00009	0.00010	0.00010	0.00010	0.00010
10000	-7	0	**0.05527**	0.00009	0.00009	0.00009	0.00009	0.00009	0.00009	0.00009
		1	**0.01910**	0.00012	0.00012	0.00012	0.00012	0.00012	0.00012	0.00012
		2	**0.00665**	0.00010	0.00010	0.00010	0.00010	0.00010	0.00010	0.00010
	-4.5	0	**0.00139**	0.00009	0.00009	0.00009	0.00009	0.00009	0.00009	0.00009
		1	**0.00090**	0.00010	0.00010	0.00010	0.00010	0.00010	0.00010	0.00010
		2	**0.00026**	0.00010	0.00010	0.00009	0.00010	0.00010	0.00010	0.00010
	-2	0	**0.00016**	0.00008	0.00008	0.00008	0.00008	0.00008	0.00008	0.00008
		1	0.00014	0.00009	0.00009	0.00009	0.00009	0.00008	0.00009	0.00009
		2	0.00011	0.00011	0.00011	0.00010	0.00011	0.00011	0.00011	0.00011

^1^The MR method is implemented by the R package *rq* [31] with the bootstrapping summary technique, when *n* = 2000, *γ*_0_ = -7 and *γ*_1_ = 0 is considered. Otherwise, the MR method is implemented by the R package *rq* [31] with the Default summary technique. The main reason is that when *n* = 2000, *γ*_0_ = -7 and *γ*_1_ = 0 is considered, the MR method cannot be implemented by the default summary technique, because the sample size *n* and the MAF are insufficiently large.

^2^The YJPT method is implemented by the R package *car* [32].

^3^The SKAT method is implemented by the R package *SKAT* [28].

^4^The D-INT, I-INT and O-INT methods are executed by the R package *RNOmni* [33].

^5^TS-INT is abbreviated from the fully adjusted two-stage INT method proposed by Sofer et al [5].

^6^FS-INT is abbreviated from the fully adjusted full-stage INT method proposed in this paper.

^†^Empirical type I error rates that are larger than or equal to 0.00016 are printed in boldface.

Moreover, we observe that when the error terms follow a normal distribution with zero mean and a smaller standard deviation of 0.01, the MR method, the D-INT and O-INT tests have inflated type I error rates, whereas the YJPT method, the SKAT test, the I-INT test and the fully adjusted two- and full-stage INT methods have a good control of type I error rates. A similar result obtained at the nominal level of 0.001 is presented in S2 Table.

**Table 2:** Empirical type I errors for the eight competing methods for each study at nominal level of 0.0001 based on error terms from a normal distribution with zero mean and a standard deviation of 0.01.

Sample	How	Con-								
Size	rare	founding	Association method							
*n*	*γ*_0_	*γ*_1_	MR^1^	YJPT^2^	SKAT^3^	D-INT^4^	I-INT^4^	O-INT^4^	TS-INT^5^	FS-INT^6^
2000	-7	0	0.00007	0.00010	0.00010	0.00000	0.00008	0.00002	0.00008	0.00008
		1	**0.03120**^†^	0.00011	0.00010	0.00000	0.00009	0.00005	0.00009	0.00009
		2	**0.00897**	0.00011	0.00010	**0.46433**	0.00010	**0.40009**	0.00011	0.00011
	-4.5	0	**0.00262**	0.00011	0.00011	0.00000	0.00011	0.00005	0.00011	0.00011
		1	**0.00326**	0.00012	0.00012	**0.00146**	0.00012	**0.00083**	0.00012	0.00012
		2	**0.00107**	0.00012	0.00011	0.00001	0.00011	0.00005	0.00011	0.00011
	-2	0	**0.00027**	0.00009	0.00009	0.00012	0.00009	0.00010	0.00009	0.00009
		1	**0.00016**	0.00011	0.00010	0.00001	0.00011	0.00006	0.00011	0.00011
		2	**0.00016**	0.00010	0.00010	0.00012	0.00010	0.00010	0.00010	0.00010
10000	-7	0	**0.05526**	0.00009	0.00009	0.00000	0.00009	0.00004	0.00009	0.00009
		1	**0.01910**	0.00012	0.00012	0.00001	0.00012	0.00007	0.00012	0.00012
		2	**0.00656**	0.00010	0.00010	**1.00000**	0.00010	**1.00000**	0.00010	0.00010
	-4.5	0	**0.00136**	0.00009	0.00009	0.00000	0.00009	0.00004	0.00009	0.00009
		1	**0.00090**	0.00010	0.00010	**0.51780**	0.00010	**0.43211**	0.00010	0.00010
		2	**0.00026**	0.00010	0.00010	**1.00000**	0.00010	**1.00000**	0.00010	0.00010
	-2	0	**0.00016**	0.00008	0.00008	0.00004	0.00008	0.00006	0.00008	0.00008
		1	0.00014	0.00009	0.00009	**0.11145**	0.00009	**0.07456**	0.00009	0.00009
		2	0.00011	0.00011	0.00011	**0.42289**	0.00011	**0.33332**	0.00011	0.00011

^1^The MR method is implemented by the R package *rq* [31] with the bootstrapping summary technique, when *n* = 2000, *γ*_0_ = -7 and *γ*_1_ = 0 is considered. Otherwise, the MR method is implemented by the R package *rq* [31] with the default summary technique. The main reason is that when *n* = 2000, *γ*_0_ = -7 and *γ*_1_ = 0 is considered, the MR method cannot be implemented by the default summary technique, because the sample size *n* and the MAF are insufficiently large.

^2^The YJPT method is implemented by the R package *car* [32].

^3^The SKAT method is implemented by the R package *SKAT* [28].

^4^The D-INT, I-INT and O-INT methods are executed by the R package *RNOmni* [33].

^5^TS-INT is abbreviated from the fully adjusted two-stage INT method proposed by Sofer et al [5].

^6^FS-INT is abbreviated from the fully adjusted full-stage INT method proposed in this paper.

^†^Empirical type I error rates that are larger than or equal to 0.00016 are printed in boldface.

Table 3 displays the simulation results of empirical type I error rates when the error terms *ε*_*i*_ with the probability of 0.99 are sampled form the normal distribution with zero mean and a standard deviation of 0.01 and with the probability of 0.01 are sampled from the normal distribution with zero mean and a standard deviation of 3. Table 3 exhibits that the YJPT method, the D-INT and O-INT tests have inflated type I error rates, while the I-INT test has deflated type I error rates. Similarly, the MR method, the SKAT test and the fully adjusted two-stage INT method have inflated type I error rates, but these methods have a good control of type I error rates when the sample size *n* is large enough or the SNP genotype has a larger MAF. In contrast with the seven existing methods, the MR method, the YJPT method, the SKAT test, the D-INT test, the I-INT test, the O-INT test and the fully adjusted full-two stage INT method, the fully adjusted full-stage INT method shows good type I error control, when the error terms involve the outliers. A similar result obtained at the nominal level of 0.001 is presented in S3 Table.

**Table 3:** Empirical type I errors for the eight competing methods for each study at nominal level of 0.0001 based on error terms involving the outliers.

Sample	How	Con-								
Size	rare	founding	Association method							
*n*	*γ*_0_	*γ*_1_	MR^1^	YJPT^2^	SKAT^3^	D-INT^4^	I-INT^4^	O-INT^4^	TS-INT^5^	FS-INT^6^
2000	-7	0	0.00007	**0.01182**	**0.01167**	**0.01514**	0.00011	**0.01486**	**0.00274**	0.00011
		1	**0.03112**^†^	**0.02277**	**0.02193**	**0.02373**	0.00003	**0.02140**	**0.00051**	0.00010
		2	**0.00905**	**0.04457**	**0.01195**	**0.02481**	0.00004	**0.02050**	**0.00130**	0.00010
	-4.5	0	**0.00257**	**0.00477**	**0.00582**	**0.00344**	0.00002	**0.00263**	0.00013	0.00010
		1	**0.00320**	**0.00754**	**0.00400**	**0.00517**	0.00001	**0.00399**	**0.00016**	0.00007
		2	**0.00103**	**0.07334**	**0.00210**	**0.00518**	0.00001	**0.00380**	**0.00016**	0.00008
	-2	0	**0.00027**	**0.00023**	**0.00035**	**0.00019**	0.00001	0.00011	0.00009	0.00010
		1	**0.00019**	**0.00476**	**0.00017**	0.00010	0.00002	0.00006	0.00010	0.00009
		2	0.00015	**0.07471**	0.00007	**0.00017**	0.00001	0.00008	0.00010	0.00009
10000	-7	0	**0.05521**	**0.01789**	**0.01778**	**0.01135**	0.00005	**0.00827**	0.00013	0.00011
		1	**0.01927**	**0.00937**	**0.00884**	**0.00530**	0.00005	**0.00440**	0.00010	0.00009
		2	**0.00659**	**0.00916**	**0.00396**	**0.01415**	0.00004	**0.01137**	0.00011	0.00009
	-4.5	0	**0.00134**	**0.00154**	**0.00159**	**0.00091**	0.00006	**0.00063**	0.00012	0.00012
		1	**0.00095**	**0.00240**	**0.00116**	**0.00146**	0.00006	**0.00107**	0.00012	0.00012
		2	**0.00030**	**0.04084**	**0.00039**	**0.00410**	0.00004	**0.00289**	0.00007	0.00008
	-2	0	**0.00019**	0.00015	**0.00016**	0.00010	0.00006	0.00008	0.00012	0.00012
		1	0.00014	**0.00277**	0.00010	0.00012	0.00006	0.00009	0.00010	0.00011
		2	0.00010	**0.03073**	0.00011	**0.00356**	0.00004	**0.00226**	0.00009	0.00009

^1^The MR method is implemented by the R package *rq* [31] with the bootstrapping summary technique, when *n* = 2000, *γ*_0_ = -7 and *γ*_1_ = 0 is considered. Otherwise, the MR method is implemented by the R package *rq* [31] with the default summary technique. The main reason is that when *n* = 2000, *γ*_0_ = -7 and *γ*_1_ = 0 is considered, the MR method cannot be implemented by the default summary technique, because the sample size *n* and the MAF are insufficiently large.

^2^The YJPT method is implemented by the R package *car* [32].

^3^The SKAT method is implemented by the R package *SKAT* [28].

^4^The D-INT, I-INT and O-INT methods are executed by the R package *RNOmni* [33].

^5^TS-INT is abbreviated from the fully adjusted two-stage INT method proposed by Sofer et al [5].

^6^FS-INT is abbreviated from the fully adjusted full-stage INT method proposed in this paper.

^†^Empirical type I error rates that are larger than or equal to 0.00016 are printed in boldface.

Table 4 reports the results of a simulation comparison on empirical type I error rates when the error terms *ε*_*i*_ are sampled from a chi-squared distribution with two degrees of freedom. Table 4 exhibits that in contrast with the YJPT method, the D-INT and O-INT tests, the MR method, the SKAT test, the I-INT test and the fully adjusted two- and full-stage INT methods can control type I errors when the sample size *n* is large enough or the SNP genotype has a larger MAF. A similar result obtained at the nominal level of 0.001 is presented in S4 Table.

**Table 4:** Empirical type I errors for the eight competing methods for each study at nominal level of 0.0001 based on non-normal error terms from a chi-squared distribution with two degrees of freedom.

Sample	How	Con-								
Size	rare	founding	Association method							
*n*	*γ*_0_	*γ*_1_	MR^1^	YJPT^2^	SKAT^3^	D-INT^4^	I-INT^4^	O-INT^4^	TS-INT^5^	FS-INT^6^
2000	-7	0	0.00007	**0.00043**	**0.00472**	**0.00051**	0.00005	**0.00032**	0.00005	0.00005
		1	**0.03722**^†^	0.00001	**0.00134**	0.00002	0.00013	0.00008	0.00014	0.00014
		2	**0.00919**	0.00000	**0.00057**	0.00000	**0.00030**	**0.00018**	**0.00032**	**0.00035**
	-4.5	0	**0.00378**	0.00013	**0.00030**	0.00014	0.00009	0.00012	0.00010	0.00010
		1	**0.00414**	0.00001	**0.00024**	0.00001	0.00013	0.00007	0.00013	0.00013
		2	**0.00133**	**0.00020**	**0.00017**	0.00006	**0.00016**	0.00011	**0.00016**	**0.00017**
	-2	0	**0.00033**	0.00009	0.00011	0.00009	0.00010	0.00010	0.00010	0.00010
		1	**0.00019**	0.00008	0.00011	0.00004	0.00010	0.00007	0.00010	0.00011
		2	0.00015	**0.00172**	0.00010	**0.00040**	0.00011	**0.00028**	0.00011	0.00011
10000	-7	0	**0.05939**	0.00005	**0.00076**	0.00006	0.00011	0.00009	0.00011	0.00011
		1	**0.02124**	0.00000	**0.00041**	0.00001	0.00012	0.00007	0.00012	0.00012
		2	**0.00854**	**0.00017**	**0.00021**	0.00004	**0.00016**	0.00011	**0.00016**	**0.00016**
	-4.5	0	**0.00188**	0.00008	**0.00016**	0.00009	0.00012	0.00011	0.00012	0.00012
		1	**0.00130**	0.00011	0.00014	0.00006	0.00013	0.00010	0.00013	0.00013
		2	**0.00035**	**0.05029**	0.00011	**0.00731**	0.00014	**0.00417**	0.00014	0.00014
	-2	0	**0.00017**	0.00007	0.00009	0.00008	0.00010	0.00008	0.00010	0.00010
		1	0.00012	**0.00241**	0.00009	**0.00061**	0.00009	**0.00035**	0.00009	0.00009
		2	0.00012	**0.09101**	0.00009	**0.01092**	0.00010	**0.00679**	0.00010	0.00010

^1^The MR method is implemented by the R package *rq* [31] with the bootstrapping summary technique, when *n* = 2000, *γ*_0_ = -7 and *γ*_1_ = 0 is considered. Otherwise, the MR method is implemented by the R package *rq* [31] with the default summary technique. The main reason is that when *n* = 2000, *γ*_0_ = -7 and *γ*_1_ = 0 is considered, the MR method cannot be implemented by the default summary technique, because the sample size *n* and the MAF are insufficiently large.

^2^The YJPT method is implemented by the R package *car* [32].

^3^The SKAT method is implemented by the R package *SKAT* [28].

^4^The D-INT, I-INT and O-INT methods are executed by the R package *RNOmni* [33].

^5^TS-INT is abbreviated from the fully adjusted two-stage INT method proposed by Sofer et al [5].

^6^FS-INT is abbreviated from the fully adjusted full-stage INT method proposed in this paper.

^†^Empirical type I error rates that are larger than or equal to 0.00016 are printed in boldface.

In summary, compared with the existing methods, the MR method, the YJPT method, the SKAT test, the D-INT test, the I-INT test, the O-INT test and the fully adjusted two-stage INT method, the fully adjusted full-stage INT approach has good performance on controlling the empirical type I error rates in our simulations, especially when the SNP genotype is uncorrelated with the covariates.

### Empirical power

**Fig 1:**
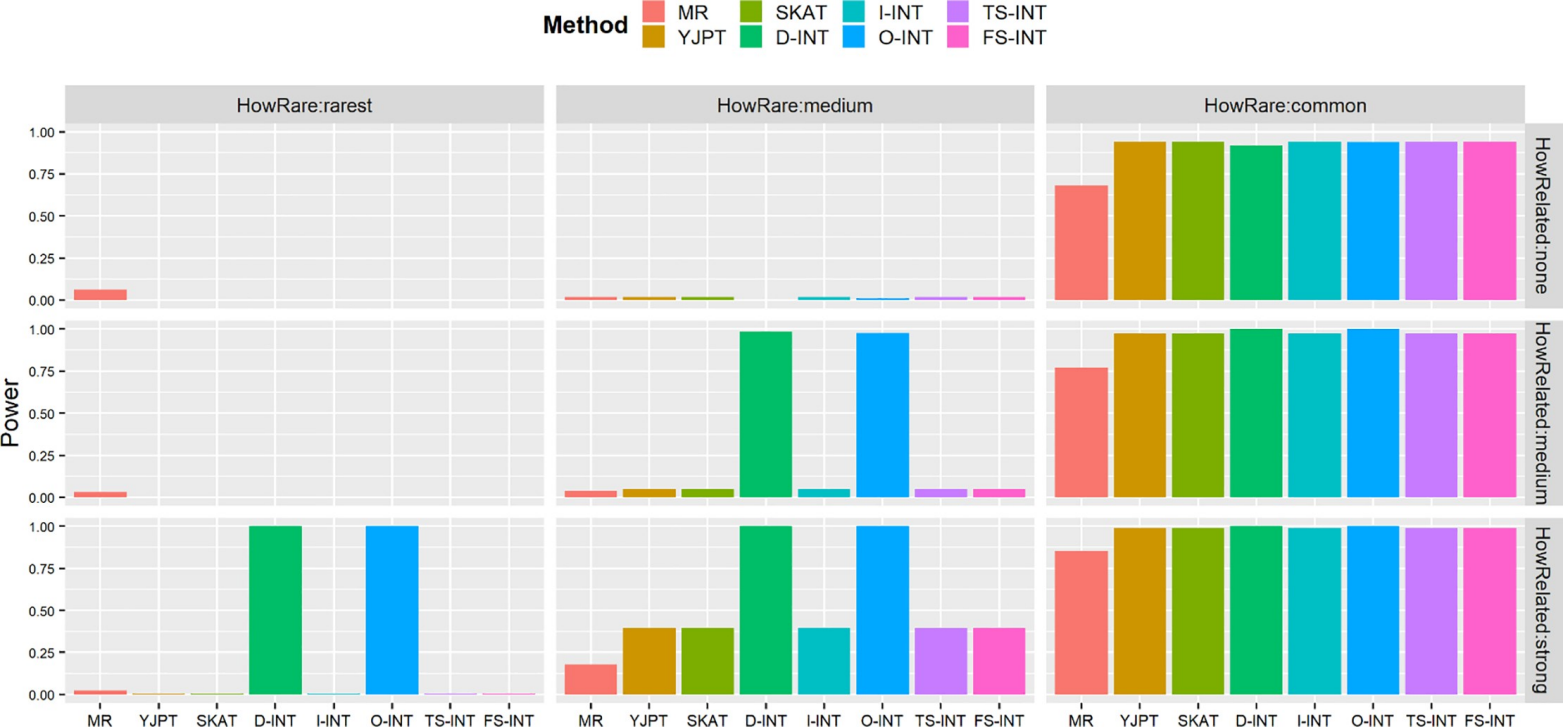
Empirical power for the eight competing methods for each study at nominal level of 0.0001 based on error terms from a normal distribution with zero mean and a standard deviation of 0.01. In the presented results, the sample size is *n* = 10000 and *β* = 0.0012. The three levels of variant frequency are considered by setting *γ*_0_ = −7 (rarest), *γ*_0_ = −4.5 (medium) and *γ*_0_ = −2 (common), respectively. The three levels of the relationship between the SNP genotype and the covariates are considered by setting *γ*_1_ = 0 (none), *γ*_1_ = 1 (medium) and *γ*_1_ = 2 (strong), respectively. The power of all of the eight competing methods is evaluated using the 2×10^5^ simulations.

**Fig 2:**
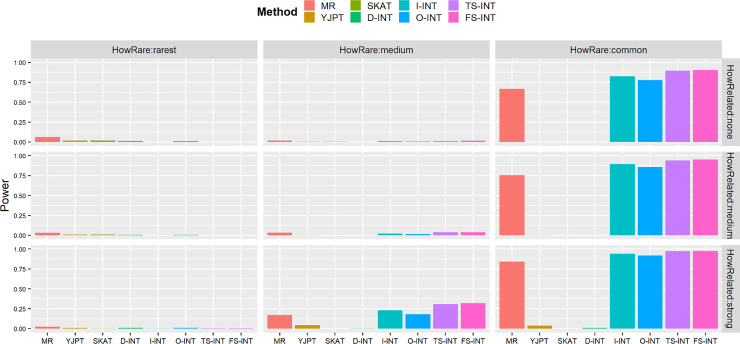
Empirical power for the eight competing methods for each study at nominal level of 0.0001 based on error terms involving the outliers. In the presented results, the sample size is *n* = 10000 and *β* = 0.0012. The three levels of variant frequency are considered by setting *γ*_0_ = −7 (rarest), *γ*_0_ = −4.5 (medium) and *γ*_0_ = −2 (common), respectively. The three levels of the relationship between the SNP genotype and the covariates are considered by setting *γ*_1_ = 0 (none), *γ*_1_ = 1 (medium) and *γ*_1_ = 2 (strong), respectively. The power of all of the eight competing methods is evaluated using the 2×10^5^ simulations.

Figs 1 and 2 and S1 Fig in S3 Appendix—S2 Fig in S4 Appendix exhibit the comparison results of empirical power based on the 2×10^5^ replicates with the sample size *n* = 10000, when the SNP (genetic) effect *β* is set by 0.0012 and the nominal level is considered by 0.0001, respectively.

Fig 1 shows that the YJPT method, the SKAT test, the I-INT test, the fully adjusted two-stage INT method (TS-INT) and the fully adjusted full-stage INT method (FS-INT) have similar power performance, when the error terms are sampled from the normal distribution with zero mean and a smaller standard deviation of 0.01. However, when the level of the variant frequency is considered to be the rarest or medium frequency (i.e., when *γ*_0_ = −7 or *γ*_0_ = −4.5) and when the level of the relationship between the SNP genotype and covariates is considered to be medium or common (i.e., when *γ*_1_ = 1 or *γ*_1_ = 2), the D-INT test and the O-INT test have better power performance in comparison with other methods, because the D-INT test and the O-INT test under the null hypothesis of no SNP (genetic) effect have inflated type I errors. Similarly, based on the same reason, false-positive power rates are obtained from the MR method when the level of the variant frequency is considered to be the rarest (i.e., when *γ*_0_ = −7).

On the basis of error terms involving the outliers, Fig 2 shows that the power rates of the fully adjusted two-stage INT method (TS-INT) and the fully adjusted full-stage INT method (FS-INT) are similar and are larger than that of the other existing methods, the MR method, the YJPT method, the SKAT test, the D-INT test, the I-INT test and the O-INT test, although the MR method, the YJPT method, the SKAT test, the D-INT test and the O-INT test under some circumstances (e.g., *γ*_0_ = −4.5 and *γ*_1_ = 2) have inflated type I errors.

The power rates based on error terms from a normal distribution with zero mean and a standard deviation of 1 are presented in S1 Fig in S3 Appendix. On the other hand, the power rates based on non-normal error terms from a chi-squared distribution with two degrees of freedom are presented in S2 Fig in S4 Appendix. They have similar results as that discussed from Figs 1 and 2.

In summary, under the alternative hypothesis, the fully adjusted full-stage INT method is as powerful as the existing methods, the MR method, the YJPT method, the SKAT test, the O-INT test and the fully adjusted two-stage INT method, when all of these eight competing methods under the null hypothesis of no SNP (genetic) effect can well control their type I errors.

## Discussion

We propose a fully adjusted full-stage INT approach for examining the association between the rare variant and the quantitative trait. The fully adjusted full-stage INT approach maintains the advantages of the fully adjusted two-stage INT approach and ameliorates the defect of the fully adjusted two-stage INT approach for rare variant association analyses. The fully adjusted two-stage INT approach proposed by Sofer et al. [5] is a special case of the fully adjusted full-stage INT approach. In comparison with the existing methods, the MR method, the YJPT method, the SKAT test, the D-INT test, the I-INT test, the O-INT test and the fully adjusted two-stage INT approach, the fully adjusted full-stage INT approach can control the type I error rates more robustly in analyzing rare variants for genetic association studies, when quantitative traits have extreme outliers or non-normality, particularly under the situation where the SNP genotype is uncorrelated with covariates.

On the other hand, we theoretically demonstrate gainful usefulness of the fully adjusted full-stage INT approach when the INT-transformed residuals, $\mathrm{RN}({\hat{\varepsilon }}_{i}),i=1,2,\cdots n,$ in the second stage of the fully adjusted two-stage INT approach, which violate the assumption requiring a normal distribution with zero mean and finite variance, are repetitively and properly transformed by the INT procedure (S1 Appendix and S2 Appendix). In addition, our simulations show that the fully adjusted full-stage INT method under the alternative hypothesis can effectively provide empirical power as that provided by the existing methods, the MR method, the YJPT method, the SKAT test, the O-INT test and the fully adjusted two-stage INT approach, when these competing methods under the null hypothesis well control the type I errors.

One of the advantages of the fully adjusted full-stage INT method is that the fully adjusted full-stage INT method can be effortlessly enforced by the R packages *glm* and *SKAT* [28]. On the basis of the gene- or region-based multiple variant test, SKAT [27, 28], the algorithm of the fully adjusted full-stage INT approach can be easily applied to examine the association between traits and variants in a specific gene or region of interest. Moreover, based on the highly-efficient rare variant association software tool, *SKAT* [28], the fully adjusted full-stage INT method, with low computational costs per step, is appropriate for a large-scale genetic association study.

However, the fully adjusted full-stage INT approach is subject to some limitations. First, the fully adjusted full-stage INT procedure is unsuitable for qualitative data. Secondly, when the error terms that are not from a normal distribution follow a heavily skewed distribution and the SNP genotype is correlated with covariates, the fully adjusted full-stage INT procedure is insufficient for normalizing quantitative data. Most of the existing INT methods suffer from the same problems. A numerical example illustrated with the corresponding explanation is given in S5 Appendix. Thus, a more effective procedure for the fully adjusted full-stage INT method is needed to be farther proposed for improving the control of empirical type I error rates, when the distribution of the error terms is highly skewed and when the SNP genotype is correlated with covariates. Thirdly, the fully adjusted full-stage INT approach cannot be directly applied to the analysis of correlated traits, because the fully adjusted full-stage INT procedure doesn’t consider the correlational relationship between the traits. Therefore, future studies are needed to extend the idea of the fully adjusted full-stage INT method for considering the correlation between traits in the analyses of correlated traits.

## Supporting information

S1 Appendix The Wald test and the SKAT test in a fully adjusted full-stage INT procedure. (PDF)

S2 Appendix The partial *F* test in a fully adjusted full-stage INT procedure. (PDF)

S3 Appendix Empirical power based on error terms from a normal distribution with zero mean and a standard deviation of 1. (PDF)

S4 Appendix Empirical power based on non-normal error terms from a chi-squared distribution with two degrees of freedom. (PDF)

S5 Appendix The fully adjusted full-stage INT approach is subject to some limitations. (PDF)

S1 Table Empirical type I errors for the eight competing methods for each study at nominal level of 0.001 based on error terms from a normal distribution with zero mean and a standard deviation of 1. (PDF)

S2 Table Empirical type I errors for the eight competing methods for each study at nominal level of 0.001 based on error terms from a normal distribution with zero mean and a standard deviation of 0.01. (PDF)

S3 Table Empirical type I errors for the eight competing methods for each study at nominal level of 0.001 based on error terms involving the outliers. (PDF)

S4 Table Empirical type I errors for the eight competing methods for each study at nominal level of 0.001 based on non-normal error terms from a chi-squared distribution with two degrees of freedom. (PDF)

S5 Table Empirical type I errors for the eight competing methods for each study at nominal level of 0.0001 based on non-normal error terms from a gamma distribution with the shape and scale parameters given by 0.1. (PDF)
