# Supplementary material for: A rank-based normalization method with the fully adjusted full-stage procedure in genetic association studies
Source: PLoS One. 2020 Jun 19;15(6):e0233847. doi: 10.1371/journal.pone.0233847 (PMC7304615; doi:10.1371/journal.pone.0233847)
Supplement: S1 Appendix — (PDF) [file pone.0233847.s001.pdf]

S1 Appendix. The Wald test and the SKAT test in a fully adjusted full-stage INT procedure

A mathematical detail for the Wald test statistic in a fully adjusted full-stage INT procedure is provided for explaining how to use the fully adjusted full-stage INT procedure for transforming the INT-transformed residuals, $\mathrm{RN}(\hat{\varepsilon}_i), i = 1, 2, \cdots n,$ in the second stage of the fully adjusted two-stage INT method in order to make the INT-transformed residuals have a normal distribution with zero mean and one standard deviation, when the INT-transformed residuals, $\mathrm{RN}(\hat{\varepsilon}_i), i = 1, 2, \cdots n,$ in the second stage don't follow the assumption of a normal distribution with zero mean and finite variance. A similar result for the SKAT test [1] based on the fully adjusted full-stage INT procedure can be obtained.

The Wald test in a fully adjusted two-stage INT procedure

In the first stage of the fully adjusted two-stage INT approach proposed by Sofer et al. [2], on the basis of the multiple linear regression $\boldsymbol{y} = \boldsymbol{X}\boldsymbol{\alpha} + \boldsymbol{g}\beta + \boldsymbol{\varepsilon}$ in Equation (1) in the text, the (raw) residuals $\hat{\boldsymbol{\varepsilon}} = \boldsymbol{y} - \boldsymbol{X}\hat{\boldsymbol{\alpha}} = (\boldsymbol{I} - \boldsymbol{H})\boldsymbol{y}$ under the null hypothesis of $H_0 : \beta = 0$ are obtained by the traits $\boldsymbol{y}$ regressed on the covariate matrix $\boldsymbol{X}$ with the estimate of the covariate effect $\hat{\boldsymbol{\alpha}} = (\boldsymbol{X}^T\boldsymbol{X})^{-1}\boldsymbol{X}^T\boldsymbol{y}$ computed according to the least squares method. Here $\boldsymbol{H} = \boldsymbol{X}(\boldsymbol{X}^T\boldsymbol{X})^{-1}\boldsymbol{X}^T$ is an $n \times n$ projection matrix and $\boldsymbol{I}$ is the identity matrix with a size of n.

In the second stage of fully adjusted two-stage INT approach, the INT-transformed

residuals, $\mathrm{RN}(\hat{\varepsilon}_i), i=1,2,\cdots n,$ is calculated based on Equation (2) in the text,

$$\mathrm{RN}(\hat{\varepsilon}_i)=\Phi^{-1}\left\{\frac{\mathrm{rank}(\hat{\varepsilon}_i)-c}{n}\right\}, c\in[0,1/2], \text{ for } i=1,2,\cdots,n.$$

Then the INT-transformed residuals, $\mathrm{RN}(\hat{\varepsilon}_i), i=1,2,\cdots n,$ are regressed on the covariates $\boldsymbol{X}$ and the SNP genotype $\boldsymbol{g}$. Precisely, in the multiple regression model, the relationship between $\mathrm{RN}(\hat{\boldsymbol{\varepsilon}}),$ $\boldsymbol{X}$ and $\boldsymbol{g}$ is given by

$$\mathrm{RN}(\hat{\boldsymbol{\varepsilon}})=\boldsymbol{X}\boldsymbol{\alpha}'+\boldsymbol{g}\beta'+\boldsymbol{\varepsilon}' \tag{S1}$$

where $\boldsymbol{\alpha}'=(\alpha_0',\alpha_1',\cdots,\alpha_{p-1}')^T$ is a $p\times 1$ vector of regression coefficients of the covariates, β' is the regression coefficient of the SNP genotype $\boldsymbol{g}$, $\boldsymbol{\varepsilon}'=(\varepsilon_1',\varepsilon_2',\cdots,\varepsilon_n')^T$ is an $n\times 1$ vector of random errors with each component independently from the normal distribution $N(0,\sigma'^2)$. Here the main interest is the focus of the SNP (genetic) effect β on the traits $\boldsymbol{y}$ in Equation (1) in the text. Equivalently, in the second stage, the main interest is to examine the null hypothesis that there is no association between the SNP genotype $\boldsymbol{g}$ and the trait component $\mathrm{RN}(\hat{\boldsymbol{\varepsilon}})$ in Equation (S1). That is to say, in the second stage, the main interest is to examine the null hypothesis of $H_0:\beta'=0,$ which is equivalently to examine the null hypothesis of $H_0:\beta=0$ in Equation (1) in the text.

The estimate of the SNP (genetic) effect β' and its variance estimate is given by

$$\hat{\beta}'=\frac{\boldsymbol{g}^T(\boldsymbol{I}-\boldsymbol{H})\mathrm{RN}(\hat{\boldsymbol{\varepsilon}})}{\boldsymbol{g}^T(\boldsymbol{I}-\boldsymbol{H})\boldsymbol{g}}$$

and

$$\mathrm{var}(\hat{\beta}') = \frac{\hat{\sigma}'^2}{\boldsymbol{g}^T(\boldsymbol{I}-\boldsymbol{H})\boldsymbol{g}}$$

where $\hat{\sigma}'^2$ is the estimate of the error variance σ'^2 in Equation (S1). The Wald test under the null hypothesis of $H_0: \beta' = 0$ is given by

$$\mathrm{W}_{\text{TS-INT}} = \frac{(\hat{\boldsymbol{\varepsilon}}')^T \boldsymbol{g}\boldsymbol{g}\hat{\boldsymbol{\varepsilon}}'}{\hat{\sigma}'^2\boldsymbol{g}^T(\boldsymbol{I}-\boldsymbol{H})\boldsymbol{g}} \tag{S2}$$

where the residuals $\hat{\boldsymbol{\varepsilon}}' = (\hat{\varepsilon}_1', \hat{\varepsilon}_2', \cdots, \hat{\varepsilon}_n')^T = \mathrm{RN}(\hat{\boldsymbol{\varepsilon}}) - \boldsymbol{X}\hat{\boldsymbol{\alpha}}' = (\boldsymbol{I}-\boldsymbol{H})\mathrm{RN}(\hat{\boldsymbol{\varepsilon}})$ is the estimate of the error terms $\boldsymbol{\varepsilon}'$ under the null hypothesis of $H_0: \beta' = 0.$ The Wald test in a fully adjusted two-stage INT procedure, $\mathrm{W}_{\text{TS-INT}} = \left\{(\hat{\boldsymbol{\varepsilon}}')^T \boldsymbol{g}\boldsymbol{g}\hat{\boldsymbol{\varepsilon}}'\right\} / \left\{\hat{\sigma}'^2\boldsymbol{g}^T(\boldsymbol{I}-\boldsymbol{H})\boldsymbol{g}\right\},$ under $H_0: \beta' = 0$ follows a chi-squared distribution with one degree of freedom.

Here it should be emphasized that the INT-transformed residuals, $\mathrm{RN}(\hat{\varepsilon}_i), i = 1, 2, \cdots n,$ in Equation (S1) in the fully adjusted two-stage INT procedure are required to follow a normal distribution with zero mean and finite variance. However, in practice, the INT-transformed residuals, $\mathrm{RN}(\hat{\varepsilon}_i), i = 1, 2, \cdots n,$ may not be sufficiently normalized by the INT processes, which in turn results in the invalid inference of the SNP (genetic) effect β' based on the Wald test in a fully adjusted two-stage INT procedure, $\mathrm{W}_{\text{TS-INT}} = \left\{(\hat{\boldsymbol{\varepsilon}}')^T \boldsymbol{g}\boldsymbol{g}\hat{\boldsymbol{\varepsilon}}'\right\} / \left\{\hat{\sigma}'^2\boldsymbol{g}^T(\boldsymbol{I}-\boldsymbol{H})\boldsymbol{g}\right\},$ in Equation (S2). Therefore, we further propose the fully adjusted full-stage INT procedure to improve the INT-transformed residuals, $\mathrm{RN}(\hat{\varepsilon}_i), i = 1, 2, \cdots n,$ in the second stage of the fully adjusted two-stage INT method, when the INT-transformed residuals violate the assumption of a normal distribution having zero mean and finite variance.

The Wald test in a fully adjusted full-stage INT procedure

In the first and second stages of the fully adjusted full-stage INT approach, processing the similar idea of the fully adjusted two-stage INT approach [2], the INT-transformed residuals, $\mathrm{RN}(\hat{\varepsilon}_i), i=1,2,\cdots n,$ in the fully adjusted full-stage INT approach are calculated based on

$$\mathrm{RN}(\hat{\varepsilon}_i)=\Phi^{-1}\left\{\frac{\mathrm{rank}(\hat{\varepsilon}_i)-0.5}{n}\right\},\ \text{for } i=1,2,\cdots,n.$$

In third stage of the fully adjusted full-stage INT approach, the INT-transformed residuals, $\mathrm{RN}(\hat{\varepsilon}_i), i=1,2,\cdots n,$ are regressed on the covariates $\boldsymbol{X}$. Precisely, in the multiple regression model, the relationship between $\mathrm{RN}(\hat{\boldsymbol{\varepsilon}})$ and $\boldsymbol{X}$ is given by

$$\mathrm{RN}(\hat{\boldsymbol{\varepsilon}})=\boldsymbol{X}\breve{\boldsymbol{\alpha}}+\breve{\boldsymbol{\varepsilon}} \qquad \text{(S3)}$$

where $\breve{\boldsymbol{\alpha}}=(\breve{\alpha}_0,\breve{\alpha}_1,\cdots,\breve{\alpha}_{p-1})^T$ is a $p\times 1$ vector of regression coefficients of the covariates and errors $\breve{\boldsymbol{\varepsilon}}=(\breve{\varepsilon}_1,\breve{\varepsilon}_2,\cdots,\breve{\varepsilon}_n)^T$ are assumed to be independently from the normal distribution $N(0,\breve{\sigma}^2)$. If the INT-transformed residuals, $\mathrm{RN}(\hat{\varepsilon}_i), i=1,2,\cdots n,$ in Equation (S3) in the fully adjusted full-stage INT approach exactly follow a standard normal distribution, then this means that there is no association between the INT-transformed residuals $\mathrm{RN}(\hat{\boldsymbol{\varepsilon}})$ and the covariates $\boldsymbol{X}$. Therefore, if one of *p*-values of the covariate effects $\breve{\boldsymbol{\alpha}}=(\breve{\alpha}_0,\breve{\alpha}_1,\cdots,\breve{\alpha}_{p-1})^T$ is less than 0.05, then this implies that the INT-transformed residuals $\mathrm{RN}(\hat{\boldsymbol{\varepsilon}})$ are associated with one element of the covariates $\boldsymbol{X}$. Thus, it means that the INT-transformed residuals $\mathrm{RN}(\hat{\boldsymbol{\varepsilon}})$ that violate the

assumption of the normal distribution with zero mean need to be further transformed for normalization through the fourth stage in the fully adjusted full-stage INT approach. On the other hand, if all of *p*-values of the covariate effects $\breve{\boldsymbol{\alpha}} = (\breve{\alpha}_0, \breve{\alpha}_1, \cdots, \breve{\alpha}_{p-1})^T$ are not less than 0.05, then this implies that the INT-transformed residuals $\mathrm{RN}(\hat{\boldsymbol{\varepsilon}})$ aren't associated with all of the covariates $\boldsymbol{X}$. Hence, it means that the INT-transformed residuals $\mathrm{RN}(\hat{\boldsymbol{\varepsilon}})$ that satisfy the assumption of the normal distribution with zero mean and finite variance are unnecessary to be further normalized. Therefore, the INT-transformed residuals $\mathrm{RN}(\hat{\boldsymbol{\varepsilon}})$ are immediately regressed by the SNP genotype $\boldsymbol{g}$ and the covariates $\boldsymbol{X}$ through the fifth stage in the fully adjusted full-stage INT approach.

In the fourth stage of the fully adjusted full-stage INT approach, the INT-transformed residuals $\mathrm{RN}(\hat{\boldsymbol{\varepsilon}})$ in the second stage that violate the assumption of the normal distribution with zero mean and finite variance are transformed by repetitively processing Steps 1-3 of the fourth stage of the fully-adjusted full-stage INT approach, until the INT-transformed residuals, $\mathrm{RN}(\hat{\varepsilon}_i), i = 1, 2, \cdots n,$ follow a standard normal distribution.

In the fifth stage of the full adjusted full-stage INT approach, as in the second stage of the full adjusted two-stage INT approach [2], the INT-transformed residuals, $\mathrm{RN}(\hat{\varepsilon}_i), i = 1, 2, \cdots n,$ are regressed on the covariates $\boldsymbol{X}$ and the SNP genotype $\boldsymbol{g}$. The relationship between $\mathrm{RN}(\hat{\boldsymbol{\varepsilon}})$, $\boldsymbol{X}$ and $\boldsymbol{g}$ is given by $\mathrm{RN}(\hat{\boldsymbol{\varepsilon}}) = \boldsymbol{X}\boldsymbol{\alpha}'' + \boldsymbol{g}\beta'' + \boldsymbol{\varepsilon}''$ where $\boldsymbol{\alpha}'' = (\alpha_0'', \alpha_1'', \cdots, \alpha_{p-1}'')^T$ is a $p \times 1$ vector

of regression coefficients of the covariates, β'' is the regression coefficient of the SNP genotype $\boldsymbol{g}$, $\boldsymbol{\varepsilon}'' = (\varepsilon_1'', \varepsilon_2'', \cdots, \varepsilon_n'')^T$ is an $n \times 1$ vector of random errors with each component independently from the normal distribution $N(0, \sigma''^2)$. Here the INT-transformed residuals $\mathrm{RN}(\hat{\varepsilon})$ in the fifth stage of the fully adjusted full-stage INT approach are sufficient to follow the standard normal distribution. Then processing the similar procedures in the second stage of the full adjusted two-stage INT approach, the Wald test $\mathrm{W}_{\text{FS-INT}} = \left\{ (\hat{\boldsymbol{\varepsilon}}'')^T \boldsymbol{g}\boldsymbol{g}\hat{\boldsymbol{\varepsilon}}'' \right\} \Big/ \left\{ \hat{\sigma}''^2 \boldsymbol{g}^T (\boldsymbol{I} - \boldsymbol{H}) \boldsymbol{g} \right\}$ based on the fully adjusted full-stage INT approach can provide valid inference for the SNP (genetic) effect β'', where $\hat{\boldsymbol{\varepsilon}}''$ and $\hat{\sigma}''^2$ are the estimates of the error terms $\boldsymbol{\varepsilon}''$ and the error variance σ''^2.

The SKAT test in a fully adjusted full-stage INT procedure

Processing the fully adjusted full-stage procedure, a similar result for the SKAT test [1] can be obtained. Let $\boldsymbol{G}$ be the $n \times k$ matrix of genotypes. Through Stages 1-4 of the fully adjusted full-stage INT procedure, the INT-transformed residuals, $\mathrm{RN}(\hat{\varepsilon}_i), i = 1, 2, \cdots n,$ in Stage 5 of the fully adjusted full-stage INT procedure follow a standard normal distribution. Then the SKAT test with variant weights equal to 1 is given by

$$(\hat{\boldsymbol{\varepsilon}}'')^T \boldsymbol{G}\boldsymbol{G}^T \hat{\boldsymbol{\varepsilon}}'' .$$

The null distribution of the SKAT test is a mixture of chi-squared distributions [1] and its *p*-value can be obtained by the R package *SKAT* [3]. The SKAT test based on the fully adjusted

full-stage INT approach can provide valid inference for the SNP (genetic) effect.

References

1. Wu MC, Lee S, Cai T, Li Y, Boehnke M, Lin X. Rare-variant association testing for sequencing data with the sequence kernel association test. American Journal of Human Genetics 2011;89:82-93.
2. Sofer T, Zheng X, Gogarten SM, Laurie CA, Grinde K, Shaffer JR, et al. A fully adjusted two-stage procedure for rank-normalization in genetic association studies. Genetic Epidemiology 2019;43:263-75.
3. Lee SS, Miropolsky L, Wu M. SNP-set (sequence) kernel association test. 2017. doi: https://cran.r-project.org/web/packages/SKAT/SKAT.pdf.
