# Supplementary material for: A rank-based normalization method with the fully adjusted full-stage procedure in genetic association studies
Source: PLoS One. 2020 Jun 19;15(6):e0233847. doi: 10.1371/journal.pone.0233847 (PMC7304615; doi:10.1371/journal.pone.0233847)
Supplement: S2 Appendix — (PDF) [file pone.0233847.s002.pdf]

S2 Appendix. The partial *F* test in a fully adjusted full-stage INT procedure

A mathematical detail for the partial *F* test in a fully adjusted full-stage INT procedure is provided for explaining why the fully adjusted full-stage INT procedure has a robust performance on control of the type I error specially under the situation in which genotypes are uncorrelated with covariates, in comparison with the fully adjusted two-stage INT procedure.

The partial F test in a fully adjusted full-stage INT procedure

Processing Stages 1-4 of the fully adjusted full-stage INT approach, in the fifth stage of the fully adjusted full-stage INT approach, the INT-transformed residuals, $\mathrm{RN}(\hat{\varepsilon}_i), i = 1, 2, \cdots n,$ which have a standard normal distribution, are immediately regressed on the SNP genotype $\boldsymbol{g}$ and the covariates $\boldsymbol{X}$. Precisely, The relationship between $\mathrm{RN}(\hat{\boldsymbol{\varepsilon}})$, $\boldsymbol{X}$ and $\boldsymbol{g}$ is given by

$$\mathrm{RN}(\hat{\boldsymbol{\varepsilon}}) = \boldsymbol{X}\boldsymbol{\alpha}'' + \boldsymbol{g}\beta'' + \boldsymbol{\varepsilon}'' \qquad \text{(S4)}$$

where $\boldsymbol{\alpha}'' = (\alpha_0'', \alpha_1'', \cdots, \alpha_{p-1}'')^T$ is a $p \times 1$ vector of regression coefficients of the covariates, β'' is the regression coefficient of the SNP genotype $\boldsymbol{g}$, $\boldsymbol{\varepsilon}'' = (\varepsilon_1'', \varepsilon_2'', \cdots, \varepsilon_n'')^T$ is an $n \times 1$ vector of random errors with each component independently from the normal distribution $N(0, \sigma''^2)$. Let $\tilde{\boldsymbol{x}}_j = (x_{1,j}, x_{2,j}, \cdots, x_{n,j})^T, j = 1, 2, \cdots, p-1.$ The regression form in Equation (S4) is re-expressed by

$$\mathrm{RN}(\hat{\boldsymbol{\varepsilon}}) = \alpha_0'' + \tilde{\boldsymbol{x}}_1\alpha_1'' + \tilde{\boldsymbol{x}}_2\alpha_2'' + \cdots + \tilde{\boldsymbol{x}}_{p-1}\alpha_{p-1}'' + \boldsymbol{g}\beta'' + \boldsymbol{\varepsilon}''. \qquad \text{(S5)}$$

According to correlation and regression theory, covariate effects $\boldsymbol{\alpha}'' = (\alpha_0'', \alpha_1'', \cdots, \alpha_{p-1}'')^T$ and the SNP (genetic) effect β'' can be written in terms of the relationship between standard deviations and partial correlation coefficients [1-3]. Precisely, the SNP (genetic) effect β'' in Equation (S5) is given by

$$\beta'' = \rho_{\mathrm{RN}(\boldsymbol{\varepsilon})\boldsymbol{g}|\tilde{\boldsymbol{x}}_1,\tilde{\boldsymbol{x}}_2,\tilde{\boldsymbol{x}}_3,\cdots,\tilde{\boldsymbol{x}}_{p-1}} \frac{\sigma_{\mathrm{RN}(\boldsymbol{\varepsilon})|\tilde{\boldsymbol{x}}_1,\tilde{\boldsymbol{x}}_2,\tilde{\boldsymbol{x}}_3,\cdots,\tilde{\boldsymbol{x}}_{p-1}}}{\sigma_{\boldsymbol{g}|\tilde{\boldsymbol{x}}_1,\tilde{\boldsymbol{x}}_2,\tilde{\boldsymbol{x}}_3,\cdots,\tilde{\boldsymbol{x}}_{p-1}}}$$

where the partial correlation coefficient $\rho_{\mathrm{RN}(\boldsymbol{\varepsilon})\boldsymbol{g}|\tilde{\boldsymbol{x}}_1,\tilde{\boldsymbol{x}}_2,\tilde{\boldsymbol{x}}_3,\cdots,\tilde{\boldsymbol{x}}_{p-1}}$ stands for the correlation between $\mathrm{RN}(\boldsymbol{\varepsilon})$ and $\boldsymbol{g}$ adjusting for covariate effects $\tilde{\boldsymbol{x}}_1, \tilde{\boldsymbol{x}}_2, \tilde{\boldsymbol{x}}_3, \cdots, \tilde{\boldsymbol{x}}_{p-1}$ as well as $\sigma_{\mathrm{RN}(\boldsymbol{\varepsilon})|\tilde{\boldsymbol{x}}_1,\tilde{\boldsymbol{x}}_2,\tilde{\boldsymbol{x}}_3,\cdots,\tilde{\boldsymbol{x}}_{p-1}}$ and $\sigma_{\boldsymbol{g}|\tilde{\boldsymbol{x}}_1,\tilde{\boldsymbol{x}}_2,\tilde{\boldsymbol{x}}_3,\cdots,\tilde{\boldsymbol{x}}_{p-1}}$ represent conditional standard deviations of $\mathrm{RN}(\boldsymbol{\varepsilon})$ and $\boldsymbol{g}$ given $\tilde{\boldsymbol{x}}_1, \tilde{\boldsymbol{x}}_2, \tilde{\boldsymbol{x}}_3, \cdots, \tilde{\boldsymbol{x}}_{p-1},$ respectively [1-3]. Correspondingly, the null hypothesis of $H_0 : \beta'' = 0$ is equal to the null hypothesis of $H_0 : \rho_{\mathrm{RN}(\boldsymbol{\varepsilon})\boldsymbol{g}|\tilde{\boldsymbol{x}}_1,\tilde{\boldsymbol{x}}_2,\tilde{\boldsymbol{x}}_3,\cdots,\tilde{\boldsymbol{x}}_{p-1}} = 0.$ The partial *F* test under the null hypothesis of $H_0 : \rho_{\mathrm{RN}(\boldsymbol{\varepsilon})\boldsymbol{g}|\tilde{\boldsymbol{x}}_1,\tilde{\boldsymbol{x}}_2,\tilde{\boldsymbol{x}}_3,\cdots,\tilde{\boldsymbol{x}}_{p-1}} = 0,$ i.e., under the null hypothesis of $H_0 : \beta'' = 0$ is given by

$$F\left(g \mid \tilde{\boldsymbol{x}}_1, \tilde{\boldsymbol{x}}_2, \tilde{\boldsymbol{x}}_3, \cdots, \tilde{\boldsymbol{x}}_{p-1}\right) = \frac{\hat{\rho}^2_{\mathrm{RN}(\boldsymbol{\varepsilon})\boldsymbol{g}|\tilde{\boldsymbol{x}}_1,\tilde{\boldsymbol{x}}_2,\tilde{\boldsymbol{x}}_3,\cdots,\tilde{\boldsymbol{x}}_{p-1}}}{1-\hat{\rho}^2_{\mathrm{RN}(\boldsymbol{\varepsilon})\boldsymbol{g}|\tilde{\boldsymbol{x}}_1,\tilde{\boldsymbol{x}}_2,\tilde{\boldsymbol{x}}_3,\cdots,\tilde{\boldsymbol{x}}_{p-1}} \Big/ (n-p-1)} \qquad \text{(S6)}$$

where $\hat{\rho}^2_{\mathrm{RN}(\boldsymbol{\varepsilon})\boldsymbol{g}|\tilde{\boldsymbol{x}}_1,\tilde{\boldsymbol{x}}_2,\tilde{\boldsymbol{x}}_3,\cdots,\tilde{\boldsymbol{x}}_{p-1}}$ is the estimate of $\rho^2{}_{\mathrm{RN}(\boldsymbol{\varepsilon})\boldsymbol{g}|\tilde{\boldsymbol{x}}_1,\tilde{\boldsymbol{x}}_2,\tilde{\boldsymbol{x}}_3,\cdots,\tilde{\boldsymbol{x}}_{p-1}}$ and the null distribution of the partial *F* test follows a *F* distribution with one and $n-p-1$ degrees of freedom [1].

Alternatively, the partial *F* test in Equation (S6) is equivalently computed by

$$F\left(g \mid \tilde{\boldsymbol{x}}_1, \tilde{\boldsymbol{x}}_2, \tilde{\boldsymbol{x}}_3, \cdots, \tilde{\boldsymbol{x}}_{p-1}\right) = T^2 = \left(\hat{\beta}'' \big/ \hat{\sigma}_{\beta''}\right)^2$$

where $T = \hat{\beta}'' / \hat{\sigma}_{\beta''}$ as well as $\hat{\beta}''$ and $\hat{\sigma}_{\beta''}$ are the estimates of β'' and its standard deviation $\sigma_{\beta''}$, respectively [1]. The null distribution of $T = \hat{\beta}'' / \hat{\sigma}_{\beta''}$ follows a *t* distribution with $n-p-1$ degrees of freedom [1].

As had been shown above, $\beta'' = 0$ is equal to $\rho_{\mathrm{RN}(\boldsymbol{\varepsilon})\boldsymbol{g}|\tilde{x}_1,\tilde{x}_2,\tilde{x}_3,\cdots,\tilde{x}_{p-1}} = 0.$ The partial correlation $\rho_{\mathrm{RN}(\boldsymbol{\varepsilon})\boldsymbol{g}|\tilde{x}_1,\tilde{x}_2,\tilde{x}_3,\cdots,\tilde{x}_{p-1}}$ is equal to zero which indicates that the SNP genotype $\boldsymbol{g}$ is uncorrelated with the INT-transformed residuals $\mathrm{RN}(\hat{\boldsymbol{\varepsilon}}).$ This in turn means that the SNP genotype $\boldsymbol{g}$ are uncorrelated with the covariates $\boldsymbol{X}$ and the traits $\boldsymbol{y},$ because the INT-transformed residuals $\mathrm{RN}(\hat{\boldsymbol{\varepsilon}})$ comprise the elements $\boldsymbol{X}$ and $\boldsymbol{y}.$ Therefore, under the null hypothesis of no association between the outcomes and the SNP (genetic) effect, the INT-transformed residuals, $\mathrm{RN}(\hat{\varepsilon}_i), i = 1, 2, \cdots n,$ in Equation (S5) can prove more valid inference for the SNP (genetic) effect, when the SNP genotype $\boldsymbol{g}$ are uncorrelated with the covariates $\boldsymbol{X}.$ Moreover, the INT-transformed residuals, $\mathrm{RN}(\hat{\varepsilon}_i), i = 1, 2, \cdots n,$ in the fifth stage of the fully adjusted full-stage INT method have sufficiently made for following a standard normal distribution, when the INT-transformed residuals, $\mathrm{RN}(\hat{\varepsilon}_i), i = 1, 2, \cdots n,$ in the second of the fully adjusted two-stage INT method don't satisfy the assumption of the normal distribution with zero mean and finite variance. Therefore, in comparison with the fully adjusted two-stage INT method, when the SNP genotype is uncorrelated with the covariates, the fully adjusted full-stage INT method, under the null hypothesis of no SNP (genetic) effect, can provide valid statistical

inference for the SNP (genetic) effect, although the distribution of the traits (or the residuals) of the regression that is not normal is heavily skewed or the traits involve the outliers.

References

1. Kleinbarum DG, Kupper LL, Nizam A, Muller KE. Applied regression analysis and other multivariable methods. 2nd Edition ed: Duxbury Press; 1988.
2. Demissie S, Cupples LA. Bias due to 2-stage residual-outcome regression analysis in genetic association studies. Genetic Epidemiology. 2011;35:592-6.
3. Che R, Motsinger-Reif AA, Brown CC. Loss of power in two-stage residual-outcome regression analysis in genetic association studies. Genetic Epidemiology. 2012;36:890-4.
