# Supplementary material for: A rank-based normalization method with the fully adjusted full-stage procedure in genetic association studies
Source: PLoS One. 2020 Jun 19;15(6):e0233847. doi: 10.1371/journal.pone.0233847 (PMC7304615; doi:10.1371/journal.pone.0233847)
Supplement: S3 Appendix — (PDF) [file pone.0233847.s003.pdf]

S3 Appendix. Empirical power based on error terms from a normal distribution with zero mean and a standard deviation of 1

Based on error terms from a normal distribution with zero mean and a standard deviation of one, S1 Fig shows that the power rates of the MR method are higher than that of other competing methods, when the level of the variant frequency is considered to be the rarest or medium frequency (i.e., when $\gamma_0 = -7$ or $\gamma_0 = -4.5$) . However, Table 1 in the text shows that the MR method under the null hypothesis of no SNP (genetic) effect has the inflation of the type I errors when the sample size *n* is not large enough or the SNP genotype has a rarer MAF. Thus, the MR method has the false-positive power rates due to its type I error inflation. On the other hand, the other seven competing methods have similar and lower power rates, because the SNP (genetic) effect that is set by $\beta = 0.0012$ is weak when the error terms are considered to be sampled from the normal distribution with zero mean and a standard deviation of one.

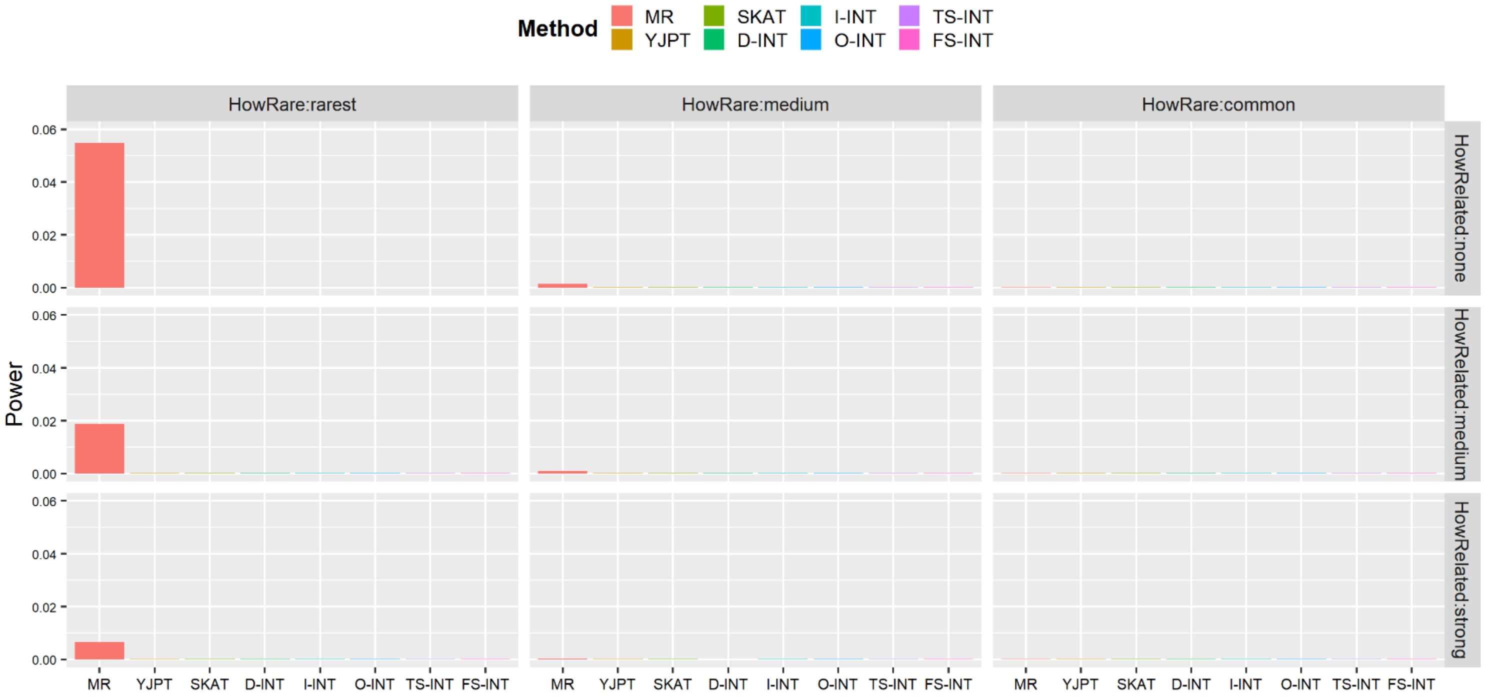

S1 Fig. Empirical power for the eight competing methods for each study at nominal level of 0.0001 based on error terms from a normal distribution with zero mean and a standard deviation of 1. In the presented results, the sample size is n = 10000 and $\beta = 0.0012$. The three levels of variant frequency are considered by setting $\gamma_0 = -7$ (rarest), $\gamma_0 = -4.5$ (medium) and $\gamma_0 = -2$ (common), respectively. The three levels of the relationship between the SNP genotype and the covariates are considered by setting $\gamma_1 = 0$ (none), $\gamma_1 = 1$ (medium) and $\gamma_1 = 2$ (strong), respectively. The power of all of the eight competing methods is evaluated using the 2×10^5 simulations.
