# Supplementary material for: A rank-based normalization method with the fully adjusted full-stage procedure in genetic association studies
Source: PLoS One. 2020 Jun 19;15(6):e0233847. doi: 10.1371/journal.pone.0233847 (PMC7304615; doi:10.1371/journal.pone.0233847)
Supplement: S4 Appendix — (PDF) [file pone.0233847.s004.pdf]

S4 Appendix. Empirical power based on non-normal error terms from a chi-squared distribution with two degrees of freedom

Based on non-normal error terms from a chi-squared distribution with two degrees of freedom, S2 Fig shows that the YJPT method, the D-INT test and the O-INT test under the some circumstances (e.g., $\gamma_0 = -4.5$ and $\gamma_1 = 2$) have higher power rates than the fully adjusted two-stage INT method (TS-INT) and the fully adjusted full-stage INT method (FS-INT), because the three methods under the null hypothesis of no SNP (genetic) effect have inflated type I errors. Similarly, the MR method has the false-positive power rates under the some circumstances (e.g., $\gamma_0 = -7$), because it under the null hypothesis of no SNP (genetic) effect has inflated type I errors.

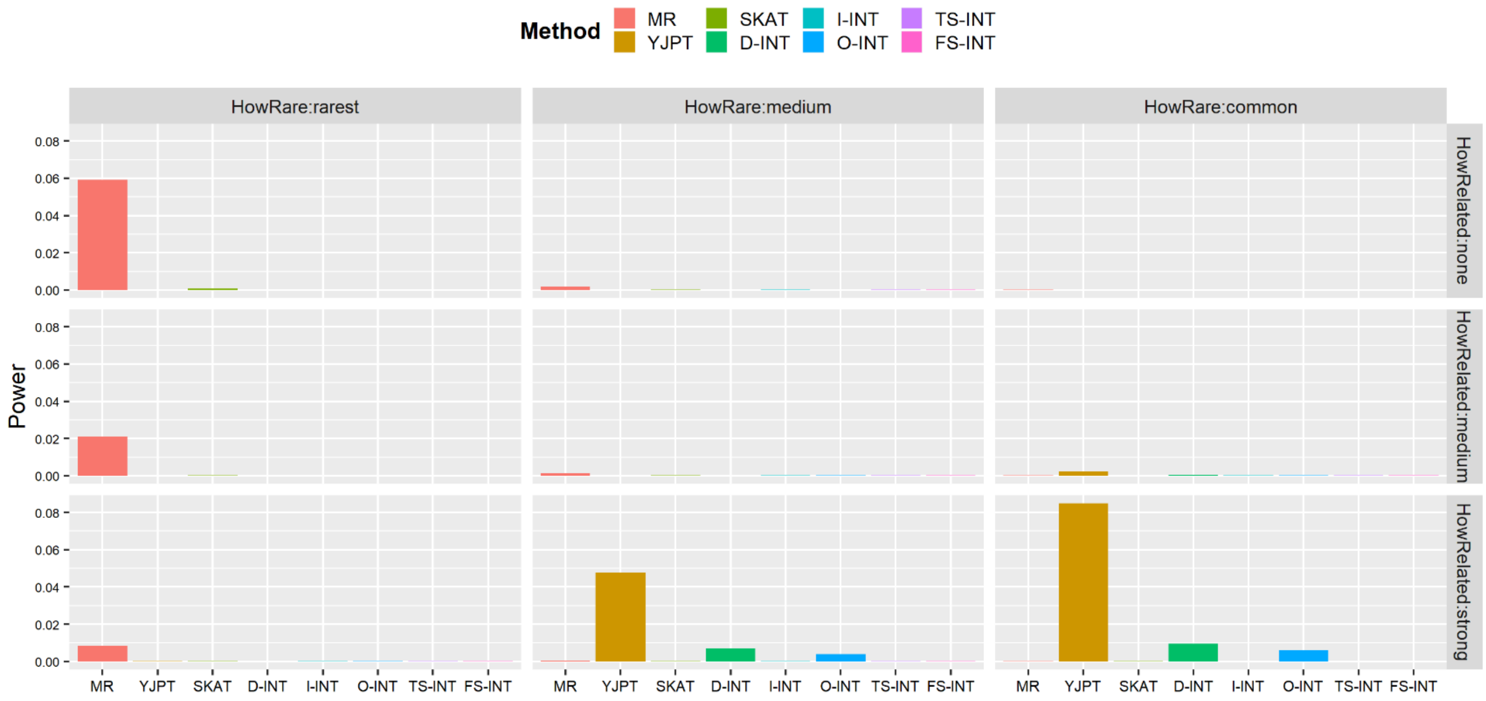

S2 Fig. Empirical power for the eight competing methods for each study at nominal level of 0.0001 based on non-normal error terms from a chi-squared distribution with two degrees of freedom. In the presented results, the sample size is n = 10000 and $\beta = 0.0012$. The three levels of variant frequency are considered by setting $\gamma_0 = -7$ (rarest), $\gamma_0 = -4.5$ (medium) and $\gamma_0 = -2$ (common), respectively. The three levels of the relationship between the SNP genotype and the covariates are considered by setting $\gamma_1 = 0$ (none), $\gamma_1 = 1$ (medium) and $\gamma_1 = 2$ (strong), respectively. The power of all of the eight competing methods is evaluated using the 2×10^5 simulations.
