# Supplementary material for: A rank-based normalization method with the fully adjusted full-stage procedure in genetic association studies
Source: PLoS One. 2020 Jun 19;15(6):e0233847. doi: 10.1371/journal.pone.0233847 (PMC7304615; doi:10.1371/journal.pone.0233847)
Supplement: S5 Appendix — (PDF) [file pone.0233847.s005.pdf]

S5 Appendix. The fully adjusted full-stage INT approach is subject to some limitations

When the error terms that are not from a normal distribution follow a heavily skewed distribution and the SNP genotype is correlated with covariates, the fully adjusted full-stage INT procedure is insufficient for normalizing quantitative data. Most of the existing INT methods suffer from the same problems. For example, following the similar set-up as those in simulation studies except that the error terms are generated from a gamma distribution with the shape and scale parameters given by 0.1, the simulation results based on n = 2000 are presented in S5 Table. The distribution of the error terms has a heave skewness and its skewness is 6.3246. S5 Table shows that the methods, the I-INT test, the O-INT test and the fully adjusted two- and full-stage INT methods, have inflated type I errors when the SNP genotype is correlated with covariates, i.e., $\gamma_1 \neq 0$. However, the fully adjusted two- and full-stage INT methods can control the type I errors when the SNP genotype is uncorrelated with covariates, i.e., $\gamma_1 = 0$ and when the SNP genotype has a sufficiently large MAF, i.e., $\gamma_0 = -2$. In contrast with the I-INT test, the O-INT test and the fully adjusted two- and full-stage INT methods, the YJPT method, the SKAT test and the D-INT test have better performance on controlling type I errors when the SNP genotype is correlated with covariates, i.e., $\gamma_1 = 2,$ and when the SNP genotype has a sufficiently large MAF, i.e., $\gamma_0 = -2.$ Thus, a more effective procedure for the fully adjusted full-stage INT method is needed to be farther proposed for improving the control of empirical type I error rates, when the distribution

of the error terms is highly skewed and when the SNP genotype is correlated with covariates.
