# Supplementary material for: A rank-based normalization method with the fully adjusted full-stage procedure in genetic association studies
Source: PLoS One. 2020 Jun 19;15(6):e0233847. doi: 10.1371/journal.pone.0233847 (PMC7304615; doi:10.1371/journal.pone.0233847)
Supplement: S2 Table — (PDF) [file pone.0233847.s007.pdf]

S2 Table. Empirical type I errors for the eight competing methods for each study at nominal level of 0.001 based on error terms from a normal distribution with zero mean and a standard deviation of 0.01.

Sample size n	How rare γ_0	Con-founding γ_1	Association method							
			MR[1]	YJPT[2]	SKAT[3]	D-INT[4]	I-INT[4]	O-INT[4]	TS-INT[5]	FS-INT[6]
2000	-7	0	**0.00526**[†]	0.00100	0.00100	0.00002	0.00094	0.00049	0.00097	0.00097
		1	**0.04241**	0.00098	0.00097	0.00003	0.00097	0.00052	0.00098	0.00098
		2	**0.01648**	0.00101	0.00100	**0.68805**	0.00099	**0.62139**	0.00099	0.00099
	-4.5	0	**0.00647**	0.00101	0.00100	0.00004	0.00100	0.00054	0.00100	0.00100
		1	**0.00787**	0.00102	0.00101	**0.01159**	0.00102	**0.00682**	0.00102	0.00102
		2	**0.00396**	0.00102	0.00101	0.00009	0.00100	0.00057	0.00100	0.00100
	-2	0	**0.00168**	0.00096	0.00094	**0.00168**	0.00094	0.00126	0.00094	0.00094
		1	0.00135	0.00096	0.00096	0.00029	0.00096	0.00063	0.00096	0.00096
		2	0.00125	0.00100	0.00099	**0.00206**	0.00099	0.00144	0.00099	0.00099
10000	-7	0	**0.07225**	0.00103	0.00103	0.00001	0.00103	0.00053	0.00103	0.00103
		1	**0.03081**	0.00099	0.00099	0.00028	0.00098	0.00065	0.00099	0.00099
		2	**0.01183**	0.00100	0.00100	**1.00000**	0.00100	**1.00000**	0.00100	0.00100
	-4.5	0	**0.00398**	0.00097	0.00097	0.00001	0.00097	0.00051	0.00097	0.00097
		1	**0.00331**	0.00102	0.00102	**0.79666**	0.00101	**0.71919**	0.00102	0.00102
		2	**0.00177**	0.00103	0.00103	**1.00000**	0.00103	**1.00000**	0.00103	0.00103
	-2	0	0.00130	0.00103	0.00102	0.00087	0.00103	0.00093	0.00104	0.00104
		1	0.00111	0.00098	0.00097	**0.34356**	0.00097	**0.25422**	0.00097	0.00097
		2	0.00112	0.00102	0.00102	**0.74488**	0.00102	**0.65025**	0.00102	0.00102

[1]The MR method is implemented by the R package *rq* [1] with the bootstrapping summary technique, when n = 2000, γ_0= -7 and $\gamma_1 = 0$ is considered. Otherwise, the MR method is implemented by the R package *rq* [1] with the default summary technique. The main reason is that when n = 2000, γ_0= -7 and $\gamma_1 = 0$ is considered, the MR method cannot be implemented by the default summary technique, because the sample size n and the MAF are insufficiently large.

[2]The YJPT method is implemented by the R package *car* [2].

[3]The SKAT method is implemented by the R package *SKAT* [3].

[4]The D-INT, I-INT and O-INT methods are executed by the R package *RNOmni* [4].

[5]TS-INT is abbreviated from the fully adjusted two-stage INT method proposed by Sofer et al [5].

[6]FS-INT is abbreviated from the fully adjusted full-stage INT method proposed in this paper.

[†]Empirical type I error rates that are larger than or equal to 0.0016 are printed in boldface.

References

1. Koenker R. Quantile regression. 2019. doi: https://cran.r-

project.org/web/packages/quantreg/quantreg.pdf.
2. Fox J, Weisberg S, Price B, Adler D, Bates D, Baud-Bovy G, et al. Companion to applied regression. 2019. doi: https://cran.r-project.org/web/packages/car/index.html.
3. Lee SS, Miropolsky L, Wu M. SNP-set (sequence) kernel association test. 2017. doi: https://cran.r-project.org/web/packages/SKAT/SKAT.pdf.
4. McCaw Z. Rank normal transformation omnibus test. 2019. doi: https://cran.r-project.org/web/packages/RNOmni/RNOmni.pdf.
5. Sofer T, Zheng X, Gogarten SM, Laurie CA, Grinde K, Shaffer JR, et al. A fully adjusted two-stage procedure for rank-normalization in genetic association studies. Genetic Epidemiology 2019;43:263-75.
