# Supplementary material for: A rank-based normalization method with the fully adjusted full-stage procedure in genetic association studies
Source: PLoS One. 2020 Jun 19;15(6):e0233847. doi: 10.1371/journal.pone.0233847 (PMC7304615; doi:10.1371/journal.pone.0233847)
Supplement: S3 Table — (PDF) [file pone.0233847.s008.pdf]

S3 Table. Empirical type I errors for the eight competing methods for each study at nominal level of 0.001 based on error terms involving the outliers.

Sample size n	How rare γ_0	Con-founding γ_1	Association method							
			MR[1]	YJPT[2]	SKAT[3]	D-INT[4]	I-INT[4]	O-INT[4]	TS-INT[5]	FS-INT[6]
2000	-7	0	**0.00520**[†]	**0.01297**	**0.01282**	**0.01597**	0.00102	**0.01576**	**0.00558**	0.00100
		1	**0.04225**	**0.02998**	**0.02898**	**0.03320**	0.00030	**0.03009**	**0.00236**	0.00100
		2	**0.01646**	**0.07410**	**0.02220**	**0.04460**	0.00036	**0.03785**	**0.00359**	0.00105
	-4.5	0	**0.00620**	**0.01321**	**0.01424**	**0.00908**	0.00020	**0.00685**	0.00113	0.00096
		1	**0.00767**	**0.01957**	**0.01013**	**0.01235**	0.00017	**0.00946**	0.00123	0.00088
		2	**0.00393**	**0.11521**	**0.00649**	**0.01471**	0.00017	**0.01073**	0.00114	0.00081
	-2	0	**0.00168**	**0.00163**	**0.00204**	0.00146	0.00018	0.00089	0.00097	0.00095
		1	0.00138	**0.01595**	0.00137	0.00108	0.00019	0.00063	0.00098	0.00095
		2	0.00123	**0.11789**	0.00080	**0.00169**	0.00018	0.00089	0.00099	0.00099
10000	-7	0	**0.07218**	**0.02921**	**0.02905**	**0.02894**	0.00058	**0.02281**	0.00111	0.00102
		1	**0.03089**	**0.01967**	**0.01878**	**0.01019**	0.00050	**0.00842**	0.00099	0.00095
		2	**0.01191**	**0.02129**	**0.00942**	**0.03075**	0.00052	**0.02451**	0.00104	0.00098
	-4.5	0	**0.00403**	**0.00510**	**0.00515**	**0.00364**	0.00053	**0.00263**	0.00101	0.00101
		1	**0.00343**	**0.00779**	**0.00415**	**0.00492**	0.00057	**0.00368**	0.00102	0.00100
		2	**0.00178**	**0.07811**	**0.00214**	**0.01465**	0.00048	**0.01017**	0.00093	0.00091
	-2	0	0.00127	0.00126	0.00128	0.00106	0.00054	0.00082	0.00100	0.00100
		1	0.00121	**0.01077**	0.00110	0.00135	0.00051	0.00093	0.00100	0.00099
		2	0.00102	**0.06455**	0.00099	**0.01740**	0.00054	**0.01112**	0.00102	0.00102

[1]The MR method is implemented by the R package *rq* [1] with the bootstrapping summary technique, when n = 2000, γ_0= -7 and $\gamma_1 = 0$ is considered. Otherwise, the MR method is implemented by the R package *rq* [1] with the default summary technique. The main reason is that when n = 2000, γ_0= -7 and $\gamma_1 = 0$ is considered, the MR method cannot be implemented by the default summary technique, because the sample size n and the MAF are insufficiently large.

[2]The YJPT method is implemented by the R package *car* [2].

[3]The SKAT method is implemented by the R package *SKAT* [3].

[4]The D-INT, I-INT and O-INT methods are executed by the R package *RNOmni* [4].

[5]TS-INT is abbreviated from the fully adjusted two-stage INT method proposed by Sofer et al [5].

[6]FS-INT is abbreviated from the fully adjusted full-stage INT method proposed in this paper.

[†]Empirical type I error rates that are larger than or equal to 0.0016 are printed in boldface.

References

1. Koenker R. Quantile regression. 2019. doi: https://cran.r-

project.org/web/packages/quantreg/quantreg.pdf.
2. Fox J, Weisberg S, Price B, Adler D, Bates D, Baud-Bovy G, et al. Companion to applied regression. 2019. doi: https://cran.r-project.org/web/packages/car/index.html.
3. Lee SS, Miropolsky L, Wu M. SNP-set (sequence) kernel association test. 2017. doi: https://cran.r-project.org/web/packages/SKAT/SKAT.pdf.
4. McCaw Z. Rank normal transformation omnibus test. 2019. doi: https://cran.r-project.org/web/packages/RNOmni/RNOmni.pdf.
5. Sofer T, Zheng X, Gogarten SM, Laurie CA, Grinde K, Shaffer JR, et al. A fully adjusted two-stage procedure for rank-normalization in genetic association studies. Genetic Epidemiology 2019;43:263-75.
