# Supplementary material for: A rank-based normalization method with the fully adjusted full-stage procedure in genetic association studies
Source: PLoS One. 2020 Jun 19;15(6):e0233847. doi: 10.1371/journal.pone.0233847 (PMC7304615; doi:10.1371/journal.pone.0233847)
Supplement: S4 Table — (PDF) [file pone.0233847.s009.pdf]

S4 Table. Empirical type I errors for the eight competing methods for each study at nominal level of 0.001 based on non-normal error terms from a chi-squared distribution with two degrees of freedom.

Sample size n	How rare γ_0	Con-founding γ_1	Association method							
			MR[1]	YJPT[2]	SKAT[3]	D-INT[4]	I-INT[4]	O-INT[4]	TS-INT[5]	FS-INT[6]
2000	-7	0	**0.00486**[†]	**0.00276**	**0.00980**	**0.00288**	0.00083	**0.00204**	0.00088	0.00091
		1	**0.04966**	0.00009	**0.00413**	0.00012	0.00111	0.00062	0.00113	0.00114
		2	**0.01701**	0.00008	**0.00221**	0.00003	**0.00184**	0.00110	**0.00190**	**0.00201**
	-4.5	0	**0.00826**	0.00120	0.00155	0.00121	0.00100	0.00114	0.00102	0.00103
		1	**0.00927**	0.00028	0.00144	0.00022	0.00113	0.00072	0.00116	0.00117
		2	**0.00446**	**0.00274**	0.00114	0.00101	0.00127	0.00114	0.00130	0.00133
	-2	0	**0.00192**	0.00100	0.00102	0.00103	0.00098	0.00103	0.00100	0.00100
		1	0.00144	0.00106	0.00103	0.00068	0.00104	0.00086	0.00105	0.00106
		2	0.00126	**0.01169**	0.00101	**0.00351**	0.00100	**0.00231**	0.00102	0.00103
10000	-7	0	**0.07712**	0.00044	**0.00287**	0.00054	0.00105	0.00082	0.00106	0.00106
		1	**0.03356**	0.00007	**0.00194**	0.00009	0.00112	0.00067	0.00113	0.00112
		2	**0.01442**	**0.00324**	0.00139	0.00103	0.00128	0.00117	0.00129	0.00131
	-4.5	0	**0.00504**	0.00077	0.00120	0.00086	0.00104	0.00095	0.00104	0.00104
		1	**0.00398**	0.00151	0.00118	0.00077	0.00111	0.00095	0.00112	0.00112
		2	**0.00192**	**0.18446**	0.00104	**0.04464**	0.00123	**0.02693**	0.00124	0.00126
	-2	0	0.00127	0.00090	0.00098	0.00091	0.00101	0.00100	0.00101	0.00101
		1	0.00118	**0.01572**	0.00095	**0.00510**	0.00102	**0.00323**	0.00103	0.00103
		2	0.00106	**0.24724**	0.00098	**0.05049**	0.00101	**0.03271**	0.00102	0.00102

[1]The MR method is implemented by the R package *rq* [1] with the bootstrapping summary technique, when $n = 2000$, $\gamma_0 = -7$ and $\gamma_1 = 0$ is considered. Otherwise, the MR method is implemented by the R package *rq* [1] with the default summary technique. The main reason is that when $n = 2000$, $\gamma_0 = -7$ and $\gamma_1 = 0$ is considered, the MR method cannot be implemented by the default summary technique, because the sample size n and the MAF are insufficiently large.
[2]The YJPT method is implemented by the R package *car* [2].
[3]The SKAT method is implemented by the R package *SKAT* [3].
[4]The D-INT, I-INT and O-INT methods are executed by the R package *RNOmni* [4].
[5]TS-INT is abbreviated from the fully adjusted two-stage INT method proposed by Sofer et al [5].
[6]FS-INT is abbreviated from the fully adjusted full-stage INT method proposed in this paper.
[†]Empirical type I error rates that are larger than or equal to 0.0016 are printed in boldface.

References

1. Koenker R. Quantile regression. 2019. doi: https://cran.r-

project.org/web/packages/quantreg/quantreg.pdf.
2. Fox J, Weisberg S, Price B, Adler D, Bates D, Baud-Bovy G, et al. Companion to applied regression. 2019. doi: https://cran.r-project.org/web/packages/car/index.html.
3. Lee SS, Miropolsky L, Wu M. SNP-set (sequence) kernel association test. 2017. doi: https://cran.r-project.org/web/packages/SKAT/SKAT.pdf.
4. McCaw Z. Rank normal transformation omnibus test. 2019. doi: https://cran.r-project.org/web/packages/RNOmni/RNOmni.pdf.
5. Sofer T, Zheng X, Gogarten SM, Laurie CA, Grinde K, Shaffer JR, et al. A fully adjusted two-stage procedure for rank-normalization in genetic association studies. Genetic Epidemiology 2019;43:263-75.
