# Supplementary material for: A rank-based normalization method with the fully adjusted full-stage procedure in genetic association studies
Source: PLoS One. 2020 Jun 19;15(6):e0233847. doi: 10.1371/journal.pone.0233847 (PMC7304615; doi:10.1371/journal.pone.0233847)
Supplement: S5 Table — (PDF) [file pone.0233847.s010.pdf]

S5 Table. Empirical type I errors for the eight competing methods for each study at nominal level of 0.0001 based on non-normal error terms from a gamma distribution with the shape and scale parameters given by 0.1.

Sample size n	How rare γ_0	Con-founding γ_1	Association method							
			MR[1]	YJPT[2]	SKAT[3]	D-INT[4]	I-INT[4]	O-INT[4]	TS-INT[5]	FS-INT[6]
2000	-7	0	0.00003	**0.01414**	**0.01408**	**0.01294**	**0.00019**	**0.01171**	**0.00080**	0.00001
		1	**0.00319**[†]	**0.00767**	**0.00762**	**0.00651**	**0.00025**	**0.00556**	**0.00078**	**0.00039**
		2	**0.00031**	**0.00348**	**0.00345**	**0.02252**	**0.00486**	**0.02025**	**0.01181**	**0.25031**
	-4.5	0	0.00006	**0.00177**	**0.00175**	**0.00119**	0.00003	**0.00087**	0.00014	0.00006
		1	0.00006	**0.00132**	**0.00130**	**0.00306**	**0.00035**	**0.00238**	**0.00127**	**0.01090**
		2	0.00002	**0.00062**	**0.00061**	0.00013	**0.00543**	**0.00383**	**0.01292**	**0.35896**
	-2	0	0.00003	**0.00026**	**0.00026**	**0.00060**	0.00001	**0.00036**	0.00010	0.00008
		1	0.00002	**0.00016**	**0.00016**	0.00006	**0.00024**	**0.00017**	**0.00093**	**0.01228**
		2	0.00001	0.00012	0.00011	0.00012	**0.00189**	**0.00124**	**0.00622**	**0.23680**

[1]The MR method is implemented by the R package *rq* [1] with the bootstrapping summary technique, when $n = 2000$, $\gamma_0 = -7$ and $\gamma_1 = 0$ is considered. Otherwise, the MR method is implemented by the R package *rq* [1] with the default summary technique. The main reason is that when $n = 2000$, $\gamma_0 = -7$ and $\gamma_1 = 0$ is considered, the MR method cannot be implemented by the default summary technique, because the sample size *n* and the MAF are insufficiently large.

[2]The YJPT method is implemented by the R package *car* [1].

[3]The SKAT method is implemented by the R package *SKAT* [2].

[4]The D-INT, I-INT and O-INT methods are executed by the R package *RNOmni* [3].

[5]TS-INT is abbreviated from the fully adjusted two-stage INT method proposed by Sofer et al [4].

[6]FS-INT is abbreviated from the fully adjusted full-stage INT method proposed in this paper.

[†]Empirical type I error rates that are larger than or equal to 0.00016 are printed in boldface.

References

1. Fox J, Weisberg S, Price B, Adler D, Bates D, Baud-Bovy G, et al. Companion to applied regression. 2019. doi: https://cran.r-project.org/web/packages/car/index.html.
2. Lee SS, Miropolsky L, Wu M. SNP-set (sequence) kernel association test. 2017. doi: https://cran.r-project.org/web/packages/SKAT/SKAT.pdf.
3. McCaw Z. Rank normal transformation omnibus test. 2019. doi: https://cran.r-project.org/web/packages/RNOmni/RNOmni.pdf.
4. Sofer T, Zheng X, Gogarten SM, Laurie CA, Grinde K, Shaffer JR, et al. A fully adjusted two-

stage procedure for rank-normalization in genetic association studies. Genetic Epidemiology 2019;43:263-75.
